# Impact of mechanical power on ICU mortality in ventilated critically ill patients: a retrospective study with continuous real-life data

**DOI:** 10.1186/s40001-024-02082-1

**Published:** 2024-10-07

**Authors:** Sara Manrique, Manuel Ruiz-Botella, Natalia Murillo, Sandra Canelles, Ivan David Victoria, Manuel Andres Samper, Oriol Plans, Laura Claverias, Mónica Magret, Federico Gordo, Oriol Roca, María Bodí

**Affiliations:** 1 Critical Care Department, Hospital Universitario Joan XXIII, Mallafré Guasch 4, 43005 Tarragona, Spain; 2 Instituto de Investigación Sanitaria Pere i Virgili, Universidad Rovira i Virgili, Tarragona, Spain; 3 Departament of Chemical Engineering, Universitat Rovira i Virgili, Tarragona, Spain; 4 Critical Care Department, Hospital Universitario del Henares, Coslada, Madrid, Spain; 5 Critical Care Department, Parc Taulí Hospital Universitari, Parc del Taulí 1, 08028 Sabadell, Spain; 6 Centro de Investigación Biomédica en Red de Enfermedades Respiratorias (CIBERES), Instituto de Salud Carlos III, Madrid, Spain; 7 Present Address: Rovira i Virgili University, Tarragona, Spain

**Keywords:** Ventilation-induced lung injury, Mechanical power, Mechanical ventilation, Protective mechanical ventilation, SARS-CoV2, Clinical information system

## Abstract

**Background:**

Over the past decade, numerous studies on potential factors contributing to ventilation-induced lung injury have been carried out. Mechanical power has been pointed out as the parameter that encloses all ventilation-induced lung injury-contributing factors. However, studies conducted to date provide data regarding mechanical power during the early hours of mechanical ventilation that may not accurately reflect the impact of power throughout the period of mechanical ventilatory support on intensive care unit mortality.

**Methods:**

Retrospective observational study conducted at a single center in Spain. Patients admitted to the intensive care unit, > *o* = 18 years of age, and ventilated for over 24 h were included. We extracted the mechanical power values throughout the entire mechanical ventilation in controlled modes period from the clinical information system every 2 min. First, we calculate the cutoff-point for mechanical power beyond which there was a greater change in the probability of death. After, the sum of time values above the safe cut-off point was calculated to obtain the value in hours. We analyzed if the number of hours the patient was under ventilation with a mechanical power above the safe threshold was associated with intensive care unit mortality, invasive mechanical ventilation days, and intensive care unit length of stay. We repeated the analysis in different subgroups based on the degree of hypoxemia and in patients with SARS CoV-2 pneumonia.

**Results:**

The cut-off point of mechanical power at with there is a higher increase in intensive care unit mortality was 18 J/min. The greater the number of hours patients were under mechanical power > 18 J/min the higher the intensive care unit mortality in all the study population, in patients with SARS CoV-2 pneumonia and in mild to moderate hypoxemic respiratory failure. The risk of death in the intensive care unit increases 0.1% for each hour with mechanical power exceeding 18 J/min. The number of hours with mechanical power > 18 J/min also affected the days of invasive mechanical ventilation and intensive care unit length of stay.

**Conclusions:**

The number of hours with mechanical power > 18 J/min is associated with mortality in the intensive care unit in critically ill patients. Continuous monitoring of mechanical power in controlled modes using an automated clinical information system could alert the clinician to this risk.

**Supplementary Information:**

The online version contains supplementary material available at 10.1186/s40001-024-02082-1.

## Background

Invasive mechanical ventilation (IMV) is a life-threatening supportive therapy does not exempt from complications [1, 2]. One of the most studied complications is ventilation-induced lung injury (VILI). Several studies have examined the causes of VILI to establish safe limits for IMV [3]. Initially, protective IMV was used for acute respiratory distress syndrome (ARDS) patients [4], and clinical practice guidelines recommended that these patients should be ventilated with a tidal volume (Vt) of 6 mL/kg predicted body weight (PBW), a plateau pressure (Pplat) below 30 cmH_2_O, and a driving pressure (DP) below 15 cmH2O [5–7]. Currently, ongoing studies are assessing if lung protective ventilation strategies are also beneficial for invasively ventilated patients who do not have lung pathology [8–10].

Some studies have shown that the Pplat and Vt are poor surrogates of pulmonary stress and strain [11]; e.g., reports indicate that strain values > 2 are the threshold that leads to lung damage [12]. In addition, other ventilatory parameters such as flow [13–15] and respiratory rate (RR) [16–18] have also been associated with increased VILI and mortality.

Gattinoni et al. [19] hypothesized that lung injury is produced by mechanical power (MP), which is the energy applied to the lung during each breath and includes most of the components that may lead to VILI. Several studies have shown that a higher MP is associated with increased incidence of VILI, longer intensive care unit (ICU) and hospital stay, higher mortality rate, and longer IMV [20–26]. A MP < 17–22 J/min has been suggested as the safe limit [20, 23–25].

Despite the aforementioned studies [20–27], the safe threshold of MP and whether MP should be considered a target in IMV or only an expression of the severity of the underlying lung damage remains unclear. More importantly, previous studies [20–27] consider single time-point MP measurements without taking into account intra-patient variations over time, which limits their conclusions.

We hypothesize that the longer the time MP remains above the recommended safe limits, the higher the ICU mortality. The aim of our study was to identify a MP cut-off point (MPcp) that better predicts ICU mortality and assess whether a longer time with MP above this MPcp is associated with higher ICU mortality.

## Methods

### Study design

Retrospective observational study conducted in a 28-bed general ICU of a tertiary university hospital between September 2015 and February 2022. All patients > *o* = 18 years of age consecutively admitted to the ICU and required IMV for more than 24 h were included in the study. Sample size was not calculated due to the large amount of collected data and the retrospective nature of the study.

### Data extraction

Data were obtained from the clinical information system (CIS, Centricity Critical Care by General Electric) and the ETL (extract, transform and load) process implemented with SQL and Python. The CIS automatically incorporates data from all upstream devices every 2 min, including IMV parameters and laboratory values. Moreover, healthcare professionals introduce all patient-related information throughout the patient care process during the ICU stay [28, 29].

We extracted demographic variables (age, sex, and body mass index [BMI]), type of patient (medical or surgical), type of ICU admission (emergency or scheduled), severity scores at 24 h of ICU admission such as Sequential Organ Failure Assessment (SOFA) [30] and Acute Physiology and Chronic Health Evaluation (APACHE) II [31], comorbidities (hypertension (HT), diabetes mellitus (DM), chronic obstructive pulmonary disease (COPD), asthma, chronic kidney disease (CKD) and heart disease, IMV days, ICU length of stay (LOS), and ICU mortality.

Peripheral oxygen saturation (SpO_2_)/inspired oxygen fraction (FiO_2_) ratio 1 h after intubation was calculated as an expression of oxygenation impairment [32]. PaO_2_ (arterial oxygen pressure)/FiO_2_ could not be calculated due to the large amount of missing data. The MP values were obtained every 2 min from the CIS.

Approach to missing data variables with missing data > 40% were excluded of database. Missing data were imputed using R-package “missForest” for statistical software R/CAN. The imputation was applied to impute the missing values of SOFA (40%), hours with DP > 15cmH2O (17%), SpO2/FiO2 1 h after admission (15%), hours with MP > 18 J/min (15%), APACHE (14%), MP median (12%), hours with Vt > 8 ml/KgPBW (7%), Vt ml/KgPBW median (7%), peak pressure (2%), IMC (0.3%), PBW (0.3%).

### Definitions

MP is defined as the amount of energy applied to the lung per unit time, measured in J/min [19]. To calculate the MP, the following formula was used [19]: MP (J/min) = 0.098 × RR × Vt × [Peak Pressure − (DP/2)], where RR is the respiratory rate, Vt is the tidal volume and DP is the driving pressure.

All respiratory variables needed to calculate the MP were automatically transferred from the ventilator to the CIS every 2 min. The respirator transferred the RR, Vt, positive end-expiratory pressure (PEEP), peak pressure, and Pplat directly; DPs were calculated by subtracting the PEEP from the Pplat. MP were only calculated when all the components of the formula where available. Pplat values were obtained only when patients were in controlled modes (Volume Control [VC] and Pressure control [PC]) and the percentage of inspiratory pause was > *o* = 10% of the respiratory cycle [33]. No manual inspiratory occlusion maneuver was performed.

Records with MP values greater than MPcp values were selected. The sum of the time interval for these selected records, converted to hours, provided the total number of hours with MP above MPcp.

### Analysis plan

**Fig. 1:**
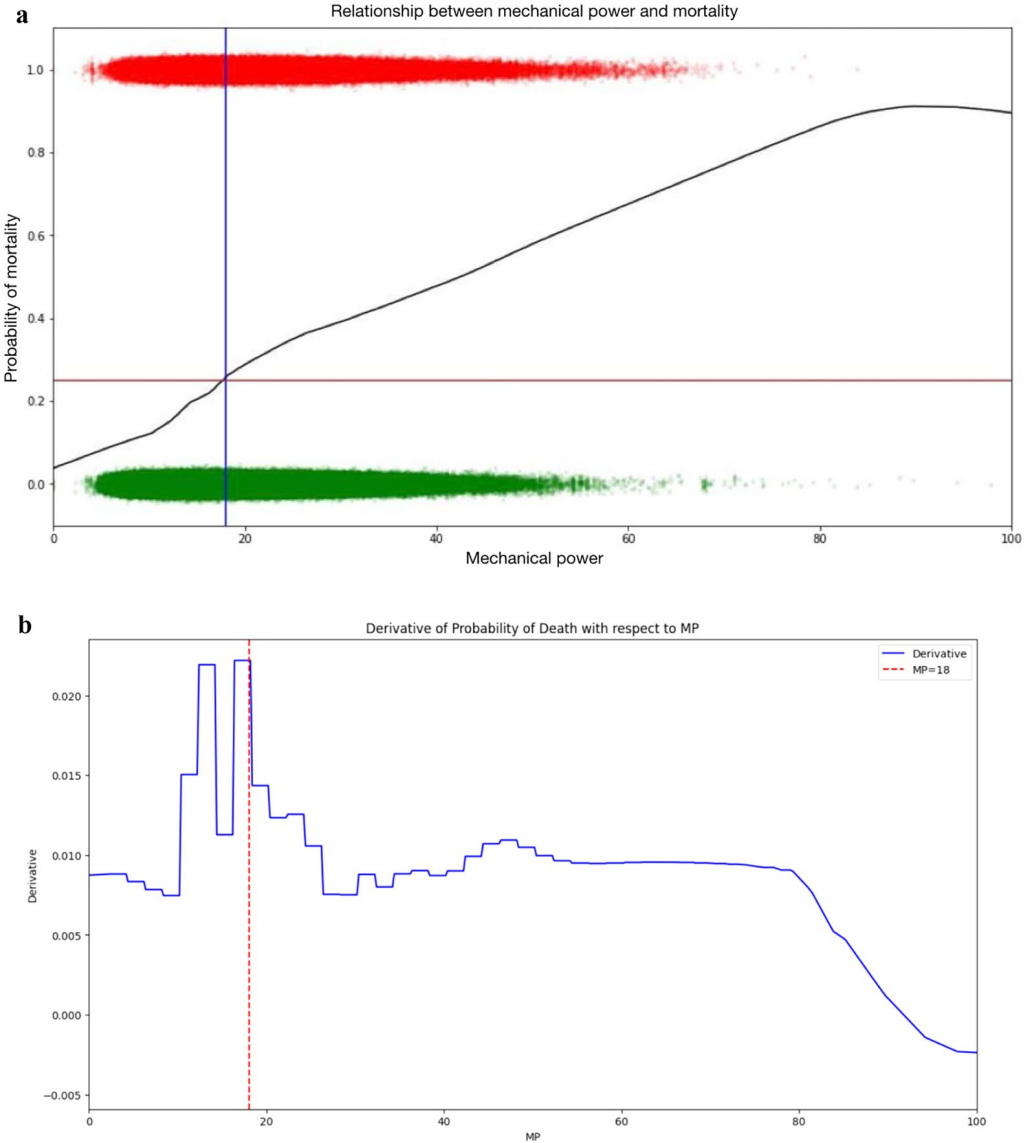
**a** The Lowess regression between the values of MP collected and the ICU mortality outcome for all the patients included in the study. The cutoff point selected was the maximum change of probability of death in the ICU between MP points. **b** The cutoff based on the derivative of probability of death in the ICU with respect to MP. The cutoff selected was the maximum change of probability of death in the ICU between MP points. The derivative is similar in values between 16.5 and 18, but maximum values by extreme decimals are actually 17.9 and 18.0

The primary outcome was finding a MP point beyond which the probability of death in the ICU increased more. We selected the threshold by making a Lowess regression between the values of MP collected and the mortality outcome for all the patients (Fig. 1A). Then we looked at the regression and selected the cutoff based on the derivative of probability of death with respect to MP (Fig. 1B). The cutoff selected was the maximum change of probability of death in the ICU between MP points.

Next, we performed a univariate analysis to identify the variables associated with higher ICU mortality. The Chi-square and the Mann–Whitney U tests were used for categorical variables and quantitative variables, respectively, as none followed a normal distribution.

Once the significant variables were identified in the univariate analysis, they were included as independent variables in a regression model (multivariate logistic regression) whose dependent variable was crude ICU mortality. Results are shown as odds ratios (OR) and 95% confidence interval (CI). For the internal model validation, the database was randomly split into two subsets: (a) a “training set” (70%) and (b) a “validation set” (30%). Model performance was examined using an accuracy test, sensitivity, specificity, positive predictive value, negative predictive value, and the area under the ROC curve (AUC). Multicollinearity was checked by calculating the variance inflation ratio (VIF).

To assess the effect of a MP above the cut-off point on IMV days and ICU LOS, Pearson correlation and simple linear regression were performed on data from survivors.

Finally, the same type of analysis was applied in the subgroup of patients with SARS CoV-2 pneumonia and subgroups based on the intensity of hypoxia [32]: SpO2/FiO2 > 355 (non-hypoxemic patients), SpO2/FiO2 between 355 and 215 (mild hypoxemia), SpO2/FiO2 between 214 and 90 (moderate hypoxemia), and SpO2/FiO2 < 90 (severe hypoxemia).

Categorical variables are presented as numbers and percentages and quantitative variables as medians and 1st and 3rd quartiles (Q1–Q3). A **p** value < 0.05 was considered significant. The R statistical platform was used for the statistical analyses (https://www.r-project.org).

## Results

### Total study population

During the study period, 10,874 patients were admitted to the ICU, from which 2623 were ventilated for more than 24 h. Seventy per cent (1826/2623) of the study population were male; median age was 64 (53–72) years, BMI 26 (24–29), SOFA 5 (4–7), and APACHE II 21 (15–25). Median SpO2/FiO2 1 h after intubation was 217 (158–279). Seventy-one per cent (1868/2623) of the study patients had a medical reason for admission. Main comorbidities were HT (30%) and DM (15%) (Table 1).

**Table 1:** Characteristics of the general population

Variable	Total population (*N* = 2623)	Died in the ICU (N = 733)	Survived in the ICU (*N* = 1890)	*p* values
General characteristics and severity of the illness				
Male, *N* (%)	1,826 (70)	514 (70)	1,312 (69)	0.76
Age (years), median (p25–75)	64 (53–72)	67 (58–74)	61 (50–71)	< 0.001
BMI, median (p25–75)	26 (24–29)	27 (24–29)	26 (24–29)	0.68
SOFA, median (p25–75)	5 (4–7)	6 (5–8)	5 (3–6)	< 0.001
APACHE II, median (p25–75)	21 (15–25)	23 (18–28)	19 (14–24)	< 0.001
Reason for admission, *N* (%)	Medical 1868 (71) Surgical 755 (29)	Medical 577 (79) Surgical 156 (21)	Medical 1291 (68) Surgical 599 (32)	< 0.001
Type of admission, *N* (%)	Urgent 2506 (95) Scheduled 117 (4)	Urgent 712 (97) Scheduled 22 (3)	Urgent 1792 (95) Scheduled 98 (5)	0.02
SpO2/FiO2 1 h within intubation, median (p25–75)	217 (158–279)	200 (142–250)	224 (163–281)	< 0.001
SARS-Cov-2, *N* (%)	277 (11)	82 (11)	195 (10)	0.56
Comorbidities				
Hypertension, *N* (%)	782 (30)	275 (38)	507 (27)	< 0.001
Diabetes, *N* (%)	386 (15)	146 (20)	240 (13)	< 0.001
Chronic heart failure, *N* (%)	119 (5)	45 (6)	74 (4)	0.02
Chronic lung disease, *N* (%)	149 (6)	65 (9)	84 (4)	< 0.001
Asthma, *N* (%)	37 (1)	7 (1)	30 (2)	0.29
Chronic kidney disease, *N* (%)	131 (5)	72 (10)	59 (3)	< 0.001
Complications and outcomes				
ICU LOS* (days), median (p25–75)	12 (6–24)*	12 (6–24)	8 (3–17)	< 0.001
ICU mortality, *N* (%)	733 (28)			
IMV days*, median (p25–75)	6 (3–15)	6 (3–15)	7 (3–15)	0.99
Tracheostomized, *N* (%)	552 (21)	95 (13)	457 (24)	< 0.001
Reintubation, *N* (%)	213 (8)	48 (6)	165 (9)	0.08
Ventilatory variables				
Hours with MP > 18 J/min, (p25–75)	34 (8–125)	44 (12–174)	31 (7–110)	< 0.001
Hours with Vt > 8 ml/KgPBW, median (p25–75)	61 (20–161)	61 (18–161)	60 (20–161)	0.51
Hours with DP > 15cmH2O, median (p25–75)	18 (3.4–89)	35.6 (7.5–143)	13 (2.5–75)	< 0.001
MP (J/min), median (p25–75)	16 (13–21)	18 (13–20)	16 (14–22)	< 0.001
Vt/KgPBW (ml/Kg), median (p25–75)	8 (7–9)	8 (7–9)	8 (7–9)	0.2
DP cmH2O, median (p25–75)	13 (11–15)	15 (12–17)	12 (10–15)	< 0.001

* Calculated using the data from survivors

*ICU* Intensive care unit, *BMI* Body Mass Index, *SOFA* Sequential Organ Failure Assessment, *APACHE* Acute Physiology and Chronic Health Evaluation, *LOS* Length of stay, *IMV* Invasive mechanical ventilation, *MP* Mechanical power, *Vt* Tidal volume, *PBW* predicted body weight

Median ICU LOS was 12 (6–24) days and median IMV 6 (3–15) days (Table 1). Twenty-one per cent (551/2623) of study patients required tracheostomy and 8% (213/2623) required reintubation during their ICU stay. Median MP—considering all IMV time in controlled modes—was 16 J/min (13–21). Crude death rate in the ICU was 28% (733/2623).

The MP threshold beyond which patients are more likely to experience increased probability of death in the ICU was similar in values between 16.5 and 18 J/min, but maximum values by extreme decimals are actually 17.9 and 18.0 J/min. Therefore, we stablished a MP = 18 J/min as the MP cut-off point (Fig. 1A and B). Median number of hours in patients ventilated at MP > 18 J/min was 34 (8–125) (Table 1).

The univariate analysis showed that the variables age, SOFA, APACHE, SpO2/FiO2 1 h after intubation, admission for medical reasons, emergency admission, HT, DM, COPD, CKD, and heart disease were associated with ICU mortality (Table 1). The number of hours with MP > 18 J/min was also associated with ICU mortality. However, no relationship was found between the number of hours with Vt > 8 ml/kgPBW and the increase in ICU mortality (Table 1). Multivariate logistic regression analysis revealed that the number of hours with MP > 18 J/min was an independent variable associated with ICU mortality (OR = 1.001; 95%CI 1.0001–1.001; AUC 0.7, e-Fig. S1 and Table 2). This means that for each hour with MP > 18 J/min, the probability of death in the ICU increases by approximately 0.1%.

**Table 2:** Multivariate logistic regression for ICU mortality

Variables	OR	CI	*p* values
General characteristics			
Age (years)	1.02	1.01–1.02	< 0.001
SOFA at admission	1.18	1.14–1.23	< 0.001
APACHE II	1.03	1.01–1.04	< 0.001
SaO2/FiO2 1 h within admission	0.99	0.99–1	0.41
Type of admission (urgent)	1.17	0.72–1.99	0.53
Reason for admission (surgical)	0.66	0.53–0.83	< 0.001
Comorbidities			
Chronic kidney disease	2.03	1.37–3.03	< 0.001
Diabetes	1.16	0.88–1.53	0.28
Chronic lung disease	1.3	0.89–1.89	0.16
Chronic heart disease	0.89	0.58–1.38	0.62
Hypertension	1.38	1.1–1.73	0.005
Respiratory variables			
Hours with MP > 18	1.001	1.0001–1.001	0.01

Overall population

*OR* Odds ratio, *CI* Confidence interval, *ICU* intensive care unit, *SOFA* Sequential Organ Failure Assessment, *APACHE* Acute Physiology and Chronic Health Evaluation, *MP* Mechanical power

Good correlation was observed between MP hours > 18 J/min and IMV days (*r* = 0.79, *p* < 0.001, e-Fig. S2). Simple linear regression showed that 62% of the variability of IVM days can be explained by the number of hours with MP > 18 J/min (*R*2 = 0.62).

Good correlation was seen between hours of MP > 18 J/min and ICU LOS (*r* = 0.73, e-Fig. S3). Simple linear regression showed that 53% of the variability in ICU LOS can be explained by the number of hours with MP > 18 J/min (*R*2 = 0.53).

### SARS-CoV-2 study patients

From the 2,623 total study patients, 277 (11%) were admitted for SARS-CoV-2 pneumonia. Table 3 shows the characteristics of this subgroup of patients. Seventy-two per cent (200/277) were male; median age was 65 years (56–71), similar to the non-COVID-19 subgroup. Median SOFA and APACHE II were 4 and 15, respectively, lower than the non-COVID subgroup (5 and 21, respectively, for SOFA and APACHE II, *p* < 0.001). COVID-19 patients had higher incidence of DM and HT, lower SpO2/FiO2 one hour after intubation, and longer ICU LOS (Table 3).

**Table 3:** Comparison between patients with and without SARS CoV-2 pneumonia

Variable	Non-COVID patients (*N* = 2346)	COVID patients (*N* = 277)	*p* values
General characteristics			
Male, *N* (%)	1626 (69)	200 (72)	0.36
Age (years) median (p25–75)	63 (52–72)	65 (56–71)	0.15
BMI, median (p25–75)	26 (24–29)	28 (26–32)	< 0.001
SOFA, median (p25–75)	5 (4–7)	4 (3–6)	< 0.001
APACHE II, median (p25–75)	21 (16–26)	15 (12–18)	< 0.001
SpO2/FiO2 1 h within intubation, median (p25–75)	238 (179–283)	131 (120–146)	< 0.001
Comorbidities			
Hypertension, *N* (%)	641 (27)	141 (51)	< 0.001
Diabetes, *N* (%)	310 (13)	76 (27)	< 0.001
Chronic heart failure, *N* (%)	96 (4)	23 (8)	0.002
Chronic lung disease, *N* (%)	134 (6)	15 (5)	0.95
Asthma, *N* (%)	26 (1)	11 (4)	0.001
Chronic kidney disease, *N* (%)	109 (5)	22 (8)	0.02
Outcomes			
ICU LOS* (days), median (p25–75)	10 (5–19)	23 (13–41)	< 0.001
ICU mortality, *N* (%)	651 (28)	82 (30)	0.56
IMV days*, median (p25–p75)	6 (3–13)	18 (9–36)	< 0.001
Ventilatory variables			
Tracheostomized, *N* (%)	450 (19)	102 (37)	< 0.001
Reintubation, *N* (%)	203 (9)	10 (4)	0.005
Hours with MP > 18 J/min, median (p25–p75)	28 (6–97)	199 (73–415)	< 0.001
Hours with TV > 8 ml/KgPBW, median (p25–p75)	59 (19–156)	85 (28–256)	< 0.001
Hours with DP > 15cmH2O, median (p25–75)	15 (3–74)	114 (14–289)	< 0.001
MP (J/min), median (p25–p75)	16 (13–20)	22 (19–29)	< 0.001
TV/KgPBW (ml/Kg), median (p25–p75)	8.2 (7.4–9)	7.9 (7.2–8.7)	0.001
DP (cmH2O), median (p25–75)	13 (10–15)	15 (12–17)	< 0.001

^*^Calculated using the data from survivors

*BMI* Body Mass Index, *SOFA* Sequential Organ Failure Assessment, *APACHE* Acute Physiology and Chronic Health Evaluation, *ICU* intensive care unit, *LOS* length of stay, *IMV* invasive mechanical ventilation, *MP* mechanical power, *Vt* Tidal volume, *PBW* predicted body weight

The comparison between non-COVID and COVID-19 patients showed that the latter presented greater alterations of variables related to protective ventilation (Table 3). No significant differences in ICU mortality was seen between the two study groups (COVID-19 30% vs. non-COVID-19 28%, *p* = 0.55).

Age, SOFA, APACHE, and the number of hours with MP > 18 J/min were associated with ICU mortality in the univariate analysis. Similar to what was observed among the general population, no significant differences were found for Vt > 8 ml/KgPBW for ICU mortality (Table 4).

**Table 4:** Univariate analysis for ICU mortality

Variables	Died in the ICU (*N* = 82)	Survived in the ICU (*N* = 195)	*p* values
General characteristics and severity of the illness			
Sex (male) *n* (%)	53 (65)	147 (75)	0.09
Age (years), median (p25–75)	69 (63–75)	63 (53–70)	< 0.001
SOFA, median (p25–75)	5 (3–6)	4 (3–6)	0.01
APACHE II, median (p25–75)	17 (14–22)	14 (11–17)	< 0.001
SaO2/FiO2 1 h within admission, median (p25–75)	129 (118–140)	132 (121–151)	0.07
Comorbidities			
Hypertension, *N* (%)	44 (54)	97 (50)	0.6
BMI, median (p25–p75)	28 (26–31)	29 (26–33)	0.1
Diabetes, *N* (%)	28 (34)	48 (25)	0.14
Chronic lung disease, *N* (%)	8 (10)	7 (4)	0.08
Asthma, *N* (%)	3 (4)	8 (4)	1
Chronic heart disease, *N* (%)	5 (6)	18 (9)	0.53
Chronic kidney disease, *N* (%)	13 (16)	9 (5)	0.004
Outcomes			
Reintubation, *N* (%)	1 (1)	9 (5)	0.29
Tracheostomized, *N* (%)	18 (22)	84 (43)	< 0.001
IMV days, median (p25–p75)	19 (13–28)	18 (8–40)	0.75
Ventilatory variables			
Hours with MP > 18, median (p25–p75)	275 (151–503)	151 (63–389)	0.004
Hours with Vt > 8 ml/KgPBW, median (p25–p75)	75 (20–163)	95 (31–273)	0.1
Hours with DP > 15cmH2O, median (p25–75)	226 (76–361)	70 (9–254)	0.001
MP (J/min), median (p25–p75)	26 (21–32)	21 (17–27)	< 0.001
Vt/KgPBW (ml/Kg), median (p25–p75)	8 (7–9)	8 (7–9)	0.91
DP (cmH2O), median (p25–75)	17 (15–20)	14 (12–16)	< 0.001

Patients with SARS CoV-2 pneumonia

*ICU* intensive care unit, *SOFA* Sequential Organ Failure Assessment, *APACHE* Acute Physiology and Chronic Health Evaluation, *BMI* Body Mass Index, *LOS* length of stay, *IMV* invasive mechanical ventilation, *MP* mechanical power, *Vt* tidal volume, *PBW* predicted body weight

The multiple regression model for ICU mortality showed that the number of hours with MP > 18 J/min was independently associated with higher mortality (OR = 1.003 (95% CI 1.001–1.004, Table 5) with an AUC 0.81 (e-Fig. S4). This means that for each hour with MP > 18 J/min the probability of death in the ICU increases 0.3%.

**Table 5:** Multivariate logistic regression for ICU mortality

Variables	OR	CI	*p* values
General characteristics			
Age	1.09	1.05–1.14	< 0.001
SOFA at admission	1.11	0.95–1.31	0.16
APACHE II	1.04	0.97–1.11	0.25
Respiratory variables			
Hours with MP > 18 J/min	1.003	1.001–1.004	< 0.001
Tracheostomized	0.08	0.03–0.23	< 0.001

Patients with SARS CoV-2 pneumonia

*OR* odds ratio, *CI* confidence interval, *SOFA* = Sequential Organ Failure Assessment, *APACHE* Acute Physiology and Chronic Health Evaluation, *MP* Mechanical power

Although a good correlation was observed between MP hours > 18 J/min and IMV days (*r* = 0.72, eFigure S5), simple linear regression showed a lower contribution of MP hours > 18 J/min to IMV days (*R*2 = 0.52) compared to that observed in non-COVID-19 patients. Similarly, the correlation between MP hours > 18 J/min and ICU LOS was good (*r* = 0.7, eFigure S6), but a lower contribution of MP hours > 18 J/min (*R*_2_ = 0.49) on ICU LOS was also observed compared to non-COVID-19 individuals.

### Analysis based on the degree of hypoxemia

There were 67 (2.5%) patients in the non-hypoxemic group (SpO2/FiO2 > 355), 1259 (48%) had mild hypoxemia (SpO2/FiO2 = 355–215), 1286 (49%) moderate hypoxemia (SpO2/FiO2 = 214–90), and 11 (0.4%) severe hypoxemia (SpO2/FiO2 < 90). Patients with higher hypoxemia were more frequently male, older, admitted for medical reason, urgent admission, higher SOFA, more comorbidities (HT,DM and COPD), and higher ICU mortality, IMV days, and ICU LOS. Patients with higher degree of hypoxemia had more hours with MP > 18 J/min and Vt > 8 ml/KgPBW, as well as higher median MP and Vt (Table 6).

**Table 6:** Demographic characteristics in different hypoxemic subgroups

Variable	No hypoxemia (*N* = 67)	Mild hypoxemia (*N* = 1259)	Moderate hypoxemia (*N* = 1286)	Severe hypoxemia (*N* = 11)	*p* values
General characteristics and severity of the illness					
Male, *N* (%)	41 (61)	836 (66)	940 (73)	9 (82)	0.001
Age (years) median (p25–75)	61 (49–68)	63 (52–72)	64 (54–72)	67 (52–76)	0.01
BMI, median (p25–75)	26 (24–28)	26 (24–29)	28 (25–31)	28 (26–30)	0.5
SOFA, median (p25–75)	4 (3–6)	5 (4–7)	6 (4–7)	6 (5–9)	< 0.001
APACHE II, median (p25–75)	19 (14–25)	20 (15–25)	21 (15–26)	21 (17–32)	0.1
Reason for admission, *N* (%)	Medical 38 (57) Surgical 29 (43)	Medical 729 (58) Surgical 530 (42)	Medical 1090 (85) Surgical 196 (15)	Medical 11 (100) Surgical 0 (0)	< 0.001
Type of admission, *N* (%)	Urgent 63 (94) Scheduled 4 (6)	Urgent 1180 (94) Scheduled 79 (6)	Urgent 1249 (97) Scheduled 37 (3)	Urgent 11 (100) Scheduled 0 (0)	< 0.001
Comorbidities					
Hypertension, *N* (%)	16 (24)	348 (28)	415 (32)	3 (27)	0.05
Diabetes, *N* (%)	7 (10)	147 (12)	230 (18)	2 (18)	< 0.001
Chronic heart failure, *N* (%)	2 (3)	48 (4)	68 (5)	1 (9)	0.2
Chronic lung disease, *N* (%)	2 (3)	55 (4)	91 (7)	1 (9)	0.02
Asthma, *N* (%)	1 (1)	18 (1)	18 (1)	0 (0)	0.9
Chronic kidney disease, *N* (%)	2 (3)	54 (4)	74 (6)	1 (9)	0.3
Complications and outcomes					
ICU LOS* (days), median (p25–75)	7 (5–15)	10 (6–21)	14 (7–29)	11 (6–16)	< 0.001
ICU mortality, *N* (%)	17 (25)	305 (24)	408 (32)	3 (27)	< 0.001
IMV days*, median (p25–p75)	3 (1–8)	5 (3–12)	8 (4–20)	7 (5–10)	< 0.001
Tracheostomized, *N* (%)	9 (13)	251 (20)	292 (23)	0 (0)	0.04
Reintubation, *N* (%)	6 (9)	110 (9)	95 (7)	2 (18)	0.4
Ventilatory variables					
Hours with MP > 18 J/min, median (p25–p75)	11 (2–101)	18 (4–64)	63 (20–199)	123 (72–200)	< 0.001
Hours with TV > 8 ml/KgPBW, median (p25–p75)	39 (6–96)	53 (18–144)	69 (21–179)	64 (26–162)	< 0.001
Hours with DP > 15 cmH2O, median (p25–75)	6 (1–25)	9 (2–51)	34 (6–143)	54 (21–84)	< 0.001
MP (J/min), median (p25–p75)	15 (11–20)	14 (12–18)	18 (15–24)	28 (20–39)	< 0.001
TV/KgPBW (ml/Kg), median (p25–p75)	8 (7–10)	8 (7–9)	8 (7–9)	8 (7–10)	< 0.001
DP (cmH2O), median (p25–75)	12 (9–14)	12 (10–14)	14 (11–16)	15 (11–17)	< 0.001

^*^Calculated using the data from survivors

*BMI* Body Mass Index, *SOFA* Sequential Organ Failure Assessment, *APACHE* Acute Physiology and Chronic Health Evaluation, *ICU* intensive care unit, *LOS* length of stay, *IMV* invasive mechanical ventilation, *MP* mechanical power, *VT* tidal volume, *PBW* predicted body weight

The univariate analysis showed a significant association between more hours with MP > 18 J/min and higher ICU mortality in the non-hypoxemic (e-Table S1), mild hypoxemic (e-Table S2), and moderate hypoxemic groups (e-Table S3), but no association was seen in patients with SpO2/FiO2 < 90 (e-Table S4). In the multivariate analysis, these differences only remain in mild and moderate hypoxemic groups (Tables 7, 8 and 9). The OR in both subgroups was 1.002, which means that the probability of death in the ICU increases 0.2% for each hour with MP > 18 J/min.

**Table 7:** Multivariate ICU mortality analysis

Variables	OR	CI	*p* values
General characteristics			
Age	1.02	0.98–1.07	0.3
SOFA at admission	1.19	0.91–1.61	0.21
APACHE II	0.98	0.9–1.07	0.68
Respiratory variables			
Hours with MP > 18 J/min	1.003	0.99–1.01	0.33
Hours with Vt > 8 ml/Kg PBW	1.003	0.99–1.01	0.19

Non hypoxemic subgroup [AUC 0.93 (0.79–1)]

*SOFA* Sequential Organ Failure Assessment, *APACHE* Acute Physiology and Chronic Health Evaluation, *MP* mechanical power, *VT* tidal volume, *PBW* predicted body weight

**Table 8:** Multivariate ICU mortality analysis

Variables	OR	CI	*p* values
General characteristics			
Age	1.01	1.002–1.02	0.02
SOFA at admission	1.19	1.13–1.27	< 0.001
APACHE II	1.05	1.03–1.07	< 0.001
Reason for admission: surgical	0.72	0.53–0.98	0.03
Type of admission: urgent	1.73	0.84–3.98	0.16
HT	1.58	1.14–2.18	0.005
CKD	1.99	1.08–3.64	0.03
Respiratory variables			
Hours with MP > 18 J/min	1.002	1.001–1.003	0.004
Hours with Vt > 8 ml/Kg PBW	0.99	0.99–1	0.06

Mildly hypoxemic patients [AUC 0.72 (0.65–0.79)]

*SOFA* Sequential Organ Failure Assessment, *APACHE* Acute Physiology and Chronic Health Evaluation, *HTA* hypertension, *DM* diabetes mellitus, *CKD* chronic kidney disease, *MP* mechanical power

**Table 9:** Multivariate ICU mortality analysis

Variables	OR	CI	*p* values
General characteristics			
Age	1.02	1.01–1.03	< 0.001
SOFA at admission	1.19	1.13–1.25	< 0.001
APACHE II	1.01	0.99–1.03	0.15
Reason for admission: surgical	0.74	0.51–1.07	0.12
Type of admission: urgent	0.76	0.37–1.64	0.47
HT	1.19	0.87–1.63	0.26
CKD	2.08	1.21–3.59	0.008
DM	1.31	0.91–1.87	0.14
CPOD	1.83	1.13–2.96	0.01
Respiratory variables			
Hours with MP > 18 J/min	1.002	1.001–1.002	< 0.001
Hours with Vt > 8 ml/Kg PBW	0.99	0.99–0.99	< 0.001

Moderate hypoxemic patients [AUC 0.68 (0.51–0.86)]

*SOFA* Sequential Organ Failure Assessment, *APACHE* Acute Physiology and Chronic Health Evaluation, *HTA* hypertension, *DM* diabetes mellitus, *CKD* chronic kidney disease, *MP* mechanical power

The Pearson correlation with IMV days between the hours with MP > 18 J/min was ranged between 0.75 and 0.79 in all subgroups (e-Figs. S7–S10). The contribution of MP hours > 18 J/min to IMV days was higher in severe hypoxemia (*R*2 = 0.83), followed by moderate hypoxemic (*R*2 = 0.62) and non-hypoxemic subgroups (*R*2 = 0.62) and, finally, followed by mild hypoxemic patients (*R*2 = 0.58).

As for the ICU LOS, Pearson correlation showed an R between 0.47 (severe hypoxemic patients, e-Fig. S14) and 0.77 in non-hypoxemic subgroups (e-Fig. S11). For mild hypoxemic patients, the correlation between hours with MP was 0.69 (e-Fig. S12) and 0.74 for moderate hypoxemic patients (e-Fig. S13). The contribution of MP hours > 18 J/min on ICU LOS was approximately 50% in all subgroups (non-hypoxemic: *R*2 = 0.58, mild hypoxemic: *R*2 = 0.47, moderate hypoxemic: *R*2 = 0.55, severe hypoxemic: *R*2 = 0.51).

## Discussion

One of the main findings in our study is that the point of MP beyond which ICU mortality increases more noticeably in critically ventilated patients is 18 J/min. Indeed, the probability of death in the ICU increases 0.1% for each hour with MP > 18 J/min in all population, and 0.2% in the mild and moderate hypoxemic subgroups and 0.3% in the COVID-19 group specifically. Furthermore, MP > 18 J/min contributes to longer IMV and ICU LOS. Surprisingly, no significant differences were found between hours of MP > 18 J/min and mortality in the group of patients with severe hypoxemia. This result could be due to the fact that in such critically ill patients, MP might not have as much influence on mortality because the severe condition of their lungs already implies a high mortality rate. However, it is important to note that the number of patients in this group is very small, so the analysis should be repeated in this subgroup when more patients are available*.*

Our results are in line with previous literature where observed a safe threshold of MP between 17 and 22 J/min [20, 23–25]. Some studies [20–27] find association between MP and ICU mortality, while another [34] found no such association. Multiple reasons may explain these discrepancies. Firstly, MPcp values vary from 17 J/min [24, 25] to 22 J/min [20], which hinders the interpretation of the results. In our study, we determined 18 J/min as a safety cut-off value, based on the relationship between ICU mortality and MP in our study population. This may differ in other cohorts and the results should be interpreted considering this. Another reason for the discrepancies may be the analysis of a "normalized" MP rather than an “absolute” MP. Several authors normalized the MP by PBW [21, 22, 26], while others by compliance [21, 22, 35] or by the amount of aerated lung visible on CT scans [22]. Zhang et al. reported that PBW-normalized MP was a better predictor of mortality than non-normalized MP [21]. However, Coppola et al. found no significant differences in mortality with respect to either MP or PBW-normalized MP, but significant differences when MP was normalized by lung compliance or CT-guided aerated lung size [22]. Contrarily, our results are based on MP absolute values analyses as in other studies [20, 23–25, 34, 36].

The different populations included and analyzed in the abovementioned studies may also lead to discrepancies. Most MP studies with different outcomes included patients with ARDS [20–22] and a strong association between MP and mortality was observed. This association has also been observed in patients without ARDS [24–27], and recently in patients with COVID-19 [23, 27]. Our results in the sub-analyses of patients with COVID-19 suggest that MP maintains a close relationship with ICU mortality, although the contribution to IMV days appears to be lower than in non-COVID-19 patients. No analyses in the ARDS subgroup (the diagnosis in our database was not reliable) were performed; however, we divided the cohort based on the degree of hypoxemia. We also found a major association between hours with MP > 18 J/min and ICU mortality in patients with mild and moderate hypoxemic subgroups. Although in the non-hypoxemic subgroup there was a significant difference in ICU mortality, this difference did not persist in the multivariate analysis. However, the number of hours with MP > 18 J/min was not an independent factor for ICU mortality in the most hypoxemic patients. These results could be explained because of the small number of patients in the severe hypoxemic subgroup.

The main strength of our study, which differentiates it from those published so far, is the continuous monitoring of MP by automatically obtaining data from the CIS. Given that IMV is a continuous process that can result in VILI at any point during IMV, it is logical to expect that the amount of time the lung is exposed to a MP above the safe cut-off point will result in an increased risk of VILI [37, 38]. MP data from the first hours or days of IMV and with spot measurements throughout the day should be interpreted with caution [21–27, 36]. Similar works have been carried out by Serpa et al. [24] and Zhu et al. in [25] with data from the MIMIC III an e-ICU database. However, the authors obtained data from the first 48 h in ICU and calculated the median between the different values obtained during 6-h intervals. Therefore, our study reflects a real-life scenario as it quantifies all the hours spent by patients under MPs above the safety cut-off points, which may lead to VILI, thus worsening the outcome.

There are some limitations to our study. Firstly, it is a single-center retrospective study, and our findings are not transferable to other populations or ICUs. Secondly, patients already intubated at ICU admission were included. Thus, IMV time prior being admitted to the ICU may have affected patient´s outcome [38]. However, most study patients were referred from the hospital ward (33%), emergency room (28%), and operating room (17%) from our hospital, so none of the patients had been on IMV for a long time prior their admission to the ICU. Only 14% of the patients were transferred from other centers. Although this “uncertain period” may be a confounding factor, if we consider that the OR for each hour of MP > 18 J/min is 1.001, its impact is marginal. Thirdly, for the calculation of the MP, the Pplat was measured during normal squared-flow ventilation by adding an inspiratory pause of 0.2–0.3 s. However, the standard method to measure Pplat with an end-inspiratory pause may underestimate the Pplat since ventilation is a dynamic process [39, 40]. This quasi-static Pplat measurement has been previously used in other studies to determine MP [33, 36]. The fact that MP could only be calculated when Pplat was available meant that we could only obtain continuous MP values for patients while they were in controlled modes, but not during the time they were in pressure support mode. However, the percentage of time our cohort spent in pressure support relative to the total mechanical ventilation time was 29% (21–34%). Therefore, in this study, self-induced lung injury (p-SILI) is not considered. There are no studies validating the use of MP in this population and other respiratory variables such as esophageal pressure or muscle pressure should also be taken into account. Future studies with continuous values of these variables should be conducted to assess p-SILI as well.

Fourthly, we applied the same MP formula for patients in volume controlled (VC) and pressure controlled (PC) ventilation, although it has been recently determined that patients in PC should be administered a different formula [41, 42]. However, 2423/2623 (92%) of our patients have never been ventilated in PC. If only patients who had been ventilated in PC are included in the analysis, the median percentage of time in PC for each patient is 14% considering the entire MV time. In the fifth place, we did not normalized MP. However, the need for normalization remains controversial.

Finally, assessing the implication of hours with MP > 18 J/min on the number of days of mechanical ventilation (MV) can be controversial, as simply having more days of MV increases the likelihood of accumulating more hours with MP > 18 J/min, making the causal relationship questionable. However, to mitigate this bias, we performed a linear regression analysis between hours with MP > 18 J/min and days of MV. The result, an *R*^2^ value of 0.62, indicates that 62% of the variability in the number of days of MV could be explained by the number of hours spent with MP > 18 J/min. Additionally, we analyzed the days of MV only in surviving patients to ensure that those who died, and consequently had fewer hours of MV, did not influence the results regarding the number of days of MV.

Our study provides evidence for the usefulness of absolute values of MP hours on the outcome of critically ill patients.

## Conclusions

The number of hours with mechanical power > 18 J/min is associated with mortality in the intensive care unit in critically ill patients. Continuous monitoring of mechanical power in controlled modes using an automated clinical information system could alert the clinician to this risk.

## Supplementary information

Supplementary Material 1

## Data Availability

The anonymized database and the data dictionary that defines each field in the set, will be available to reviewers if they consider necessary prior confidentiality agreement. Information contact: Sara Manrique, Critical Care Department—Hospital Universitario de Tarragona Joan XXIII, Tarragona, Spain, whose mail is smanriquemoreno@gmail.com.

## Abbreviations

ICU: Intensive care unit
J: Jules
min: Minutes
IMV: Invasive mechanical ventilation
VILI: Ventilation-induced lung injury
ARDS: Acute respiratory distress syndrome
Vt: Tidal volume
PBW: Predictive body weight
Pplat: Plateau pressure
DP: Driving pressure
RR: Respiratory rate
MP: Mechanical power
MPcp: Mechanical power cut-off point
CIS: Clinical information system
ETL: Extract, transform, and load
BMI: Body mass index
SOFA: Sequential Organ Failure Assessment
APACHE: Acute Physiology and Chronic Health Evaluation
HT: Hypertension
DM: Diabetes mellitus
COPD: Chronic obstructive pulmonary disease
CKD: Chronic kidney disease
LOS: Length of stay
SpO2: Peripheral oxygen saturation
FiO2: Inspired oxygen fraction
PaO_2_: Arterial oxygen pressure
OR: Odds ratio
CI: Confidence interval
AUC: Area under the ROC curve
VC: Volume controlled
PC: Pressure controlled
