# Supplementary material for: Impact of mechanical power on ICU mortality in ventilated critically ill patients: a retrospective study with continuous real-life data
Source: Eur J Med Res. 2024 Oct 7;29:491. doi: 10.1186/s40001-024-02082-1 (PMC11457382; doi:10.1186/s40001-024-02082-1)
Supplement: Supplementary file 1 — Supplementary Material 1 [file 40001_2024_2082_MOESM1_ESM.docx]

**Impact of mechanical power on ICU mortality in ventilated critically ill patients. A retrospective study with continuous real-life data**

*Manrique S^1,2#*^, Ruiz-Botella M^1,3*^, Natalia M^1^, Canelles S^1^, Victoria ID^1^, Samper MA^1,^ Plans O^1^, Claverias L^1^ ,Magret M^1^, Gordo F^4^, Roca O^5,6^ and Bodí M^1,2,6^.*

**Supplementary material**


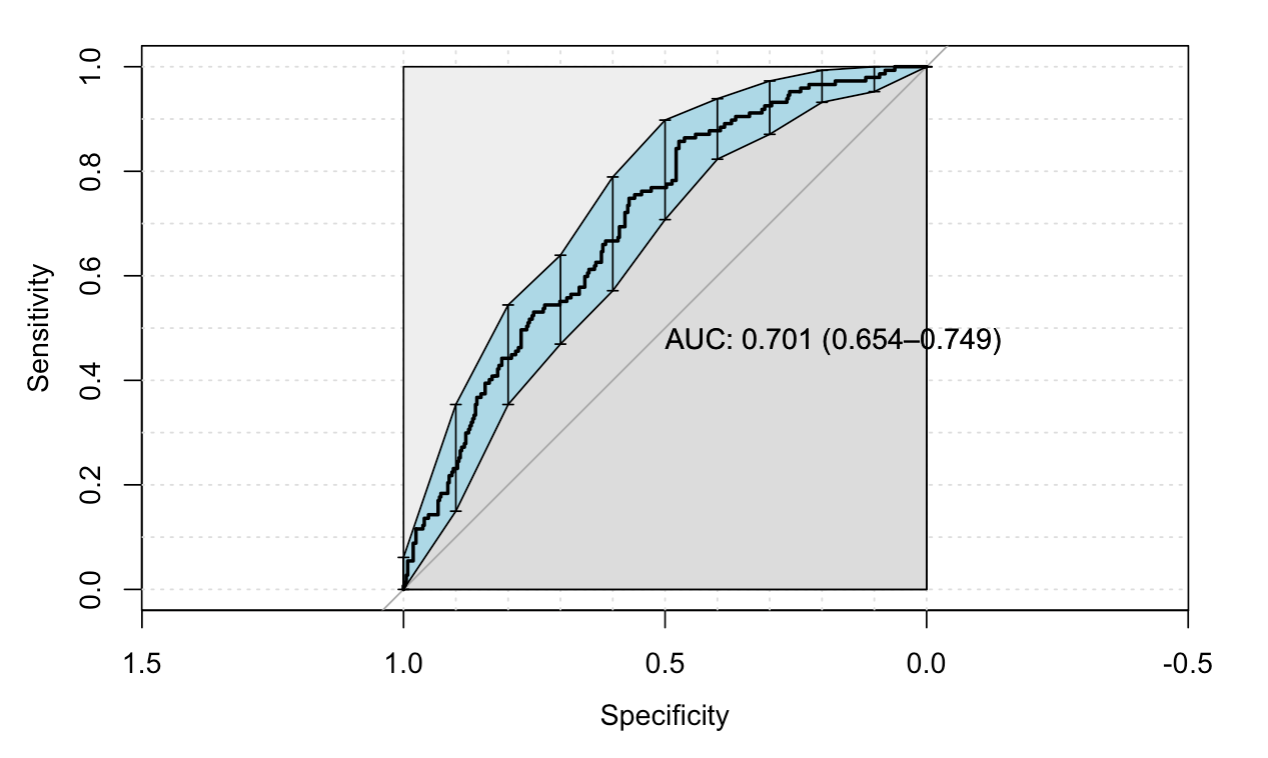
e-Figure S1. Area under the curve multivariate model of ICU mortality. Total study population

e-Figure S2. Pearson correlation between hours of mechanical power >18J/min and invasive mechanical ventilation days

*
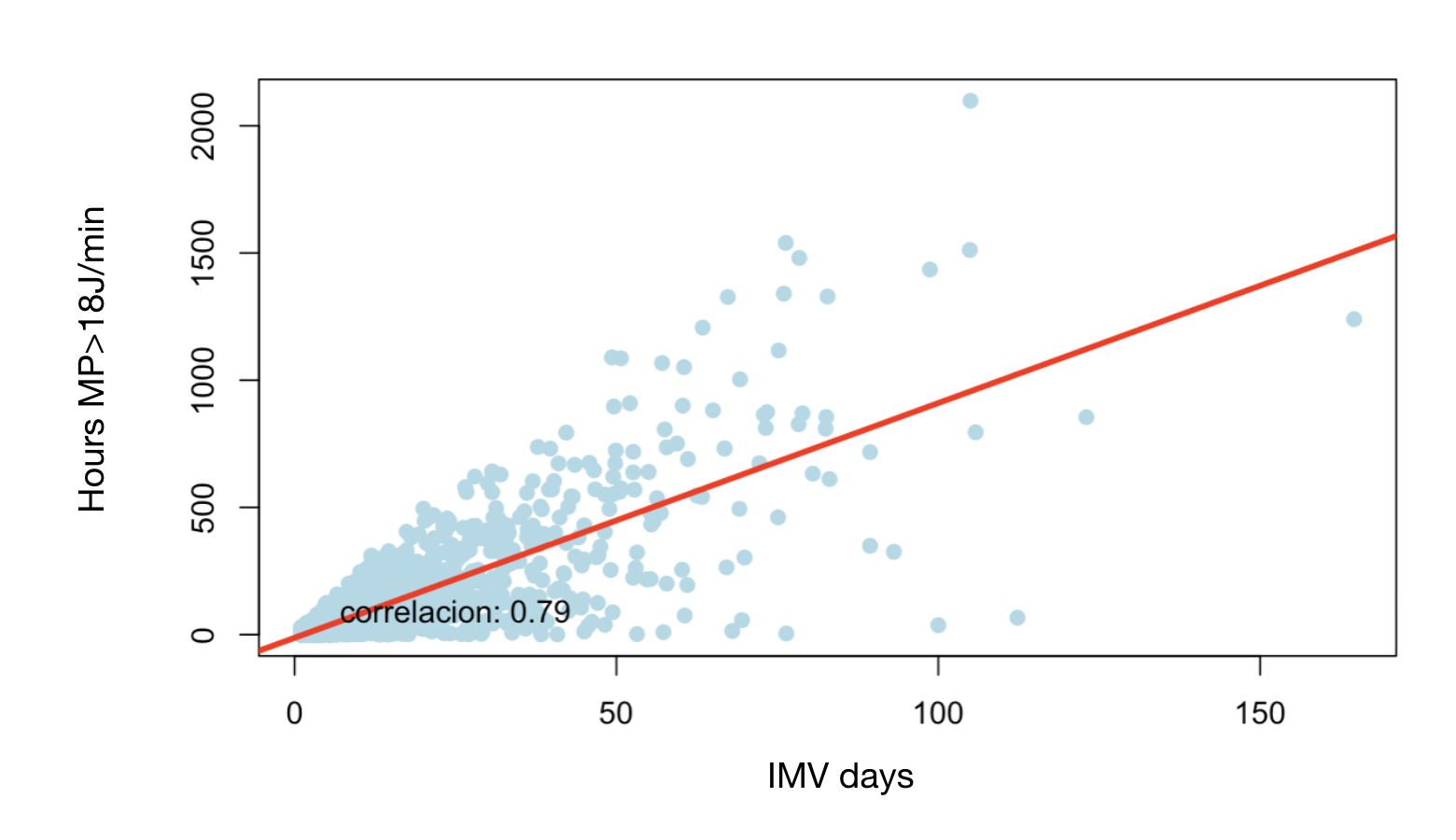
*

MP = Mechanical Power; IMV = Invasive Mechanical Ventilation

e-Figure S3. Pearson correlation between hours of mechanical power >18J/min and intensive care unit length *
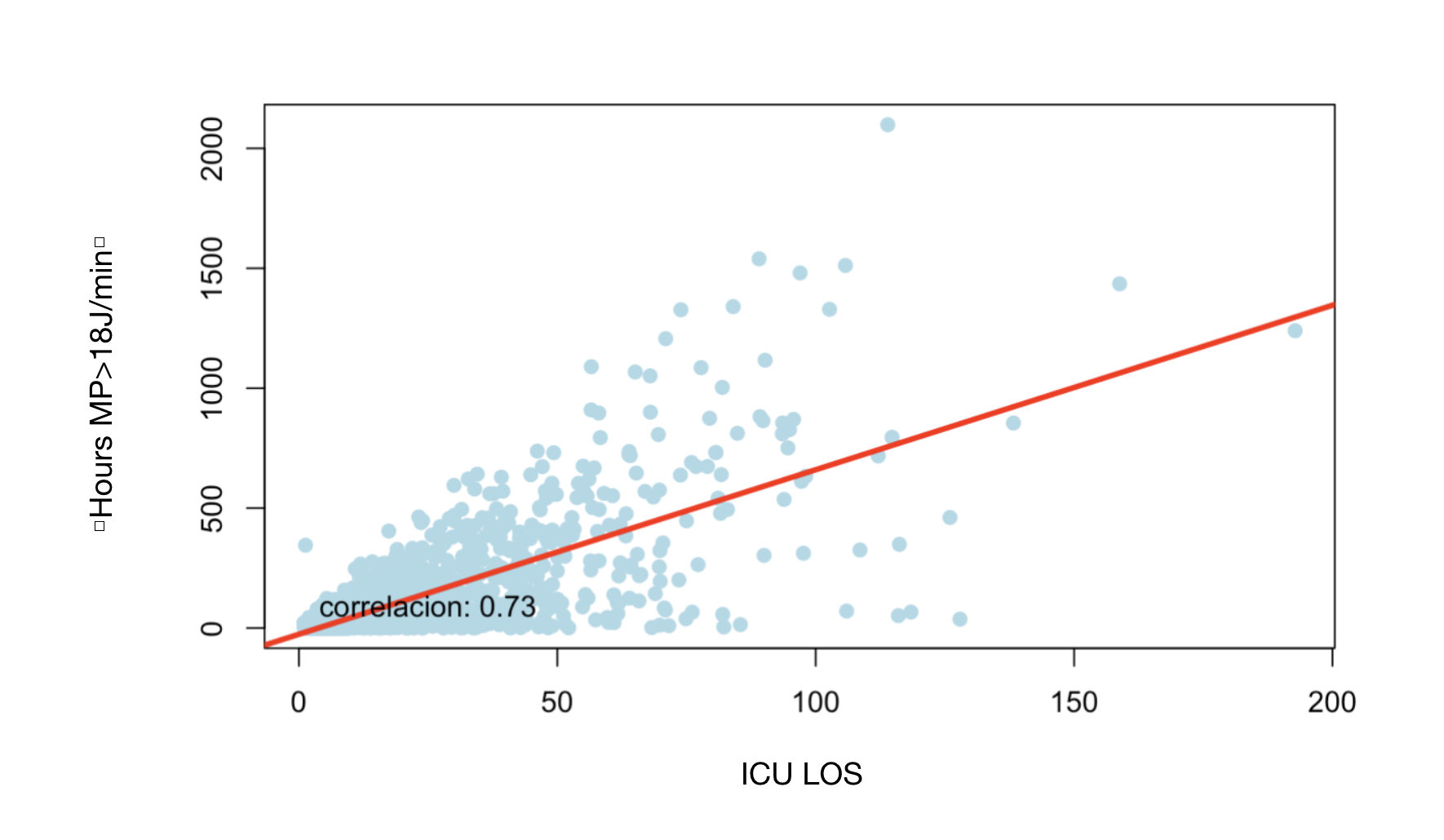
*of stay

MP = mechanical power, ICU LOS= intensive care unit length of stay

e-Figure S4. Area under the curve multivariate model of ICU mortality. Patients with SARS CoV 2 pneumonia
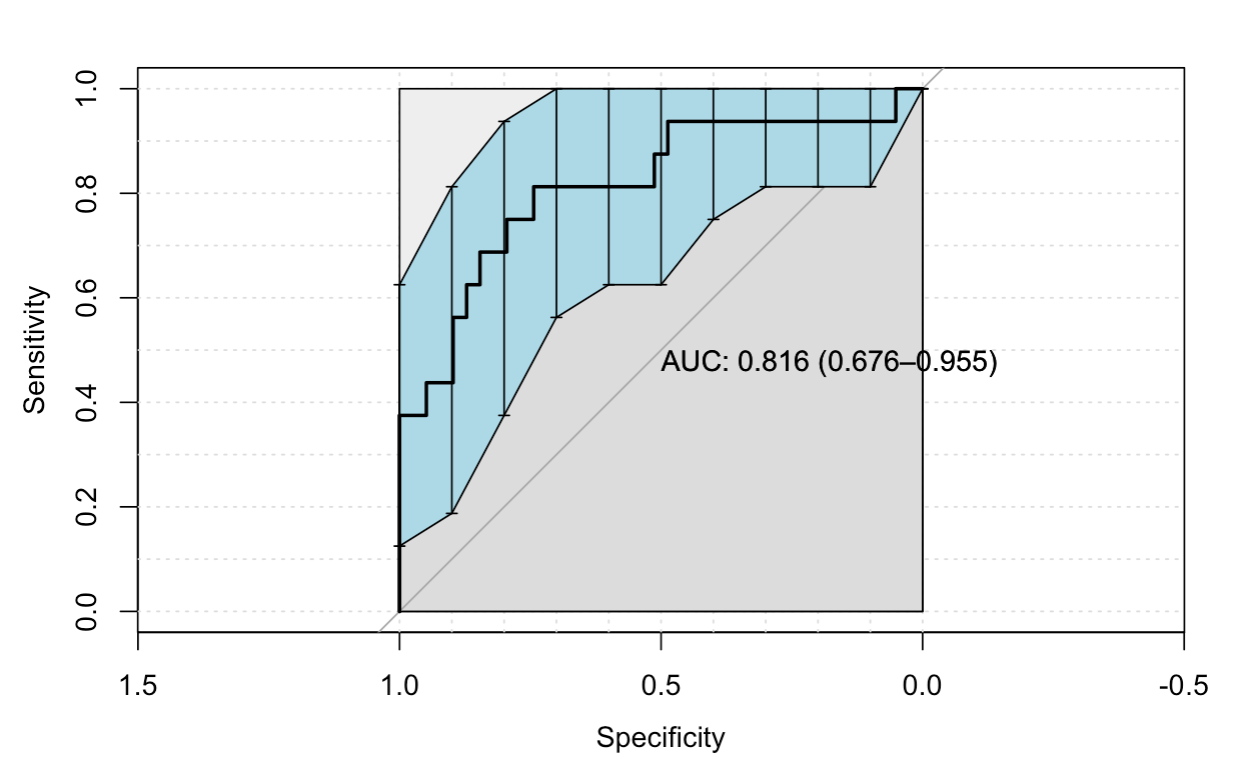


e-Figure S5. Pearson correlation between hours of mechanical power >18 J/min and invasive mechanical *
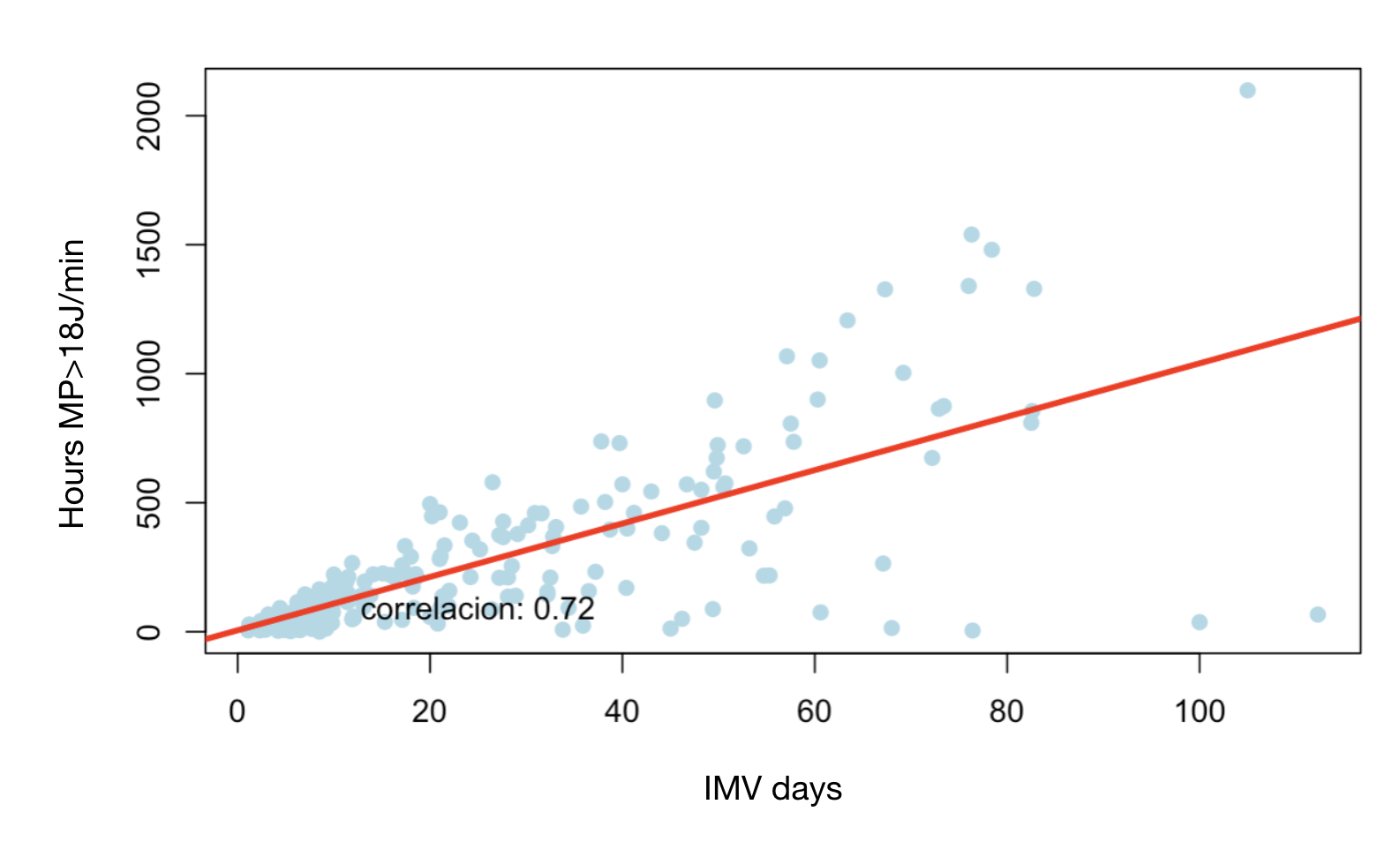
*ventilation days. Patients with SARS CoV-2 *pneumonia*

MP = mechanical power, IMV= invasive mechanical ventilation

e-Figure S6. Pearson correlation between hours of mechanical power >18 J/min and intensive care unit days
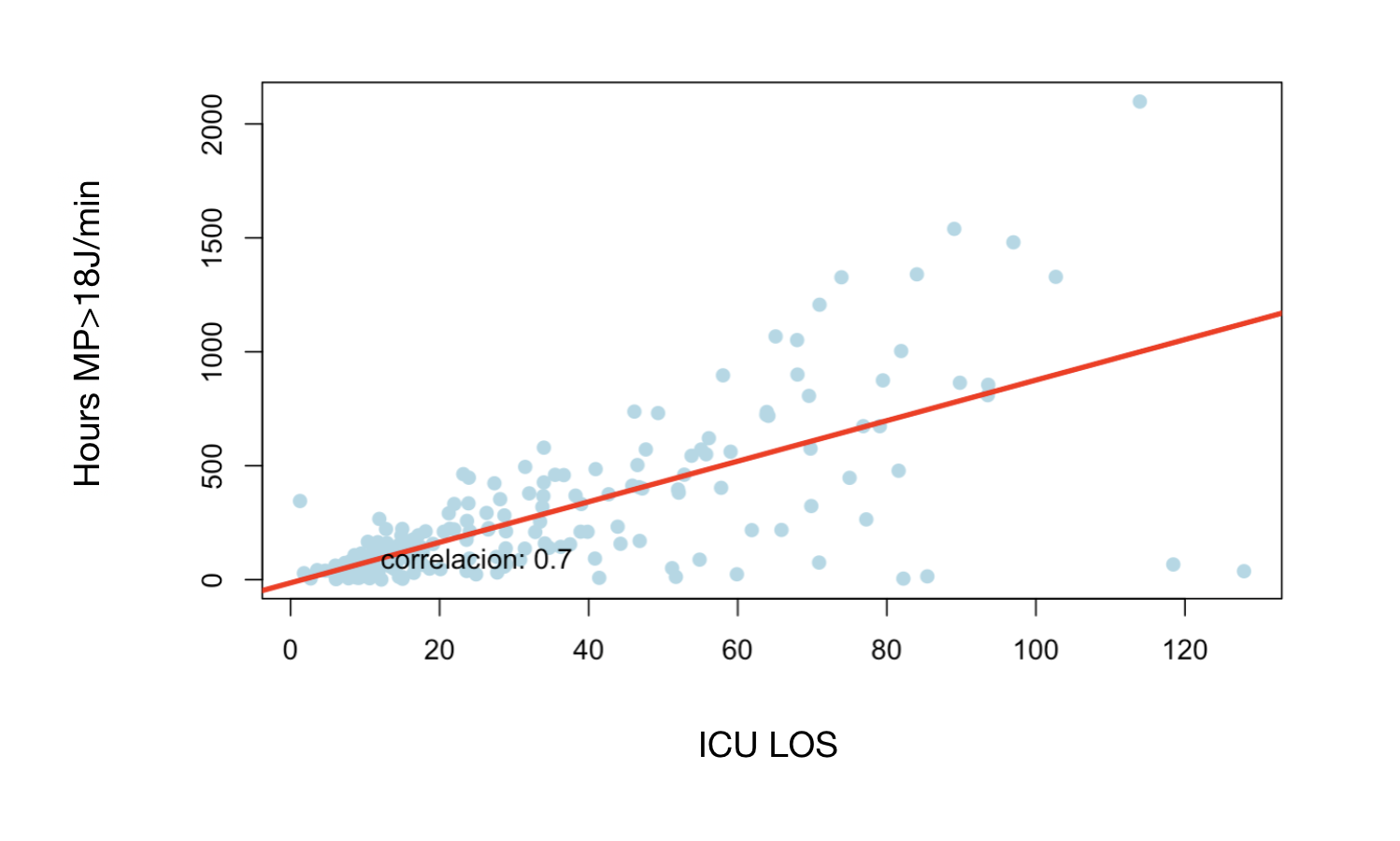
of stay

MP = mechanical power, ICU LOS = intensive care unit length of stay

e-Table S1. Univariate ICU mortality analysis- Non-hypoxemic patients

| **Variable** | **Died in the ICU**  (n=17) | **Survived in the ICU**  (n=50) | **P values** |
| --- | --- | --- | --- |
| **General characteristics and severity of the illness** | | | |
| **Male, n (%)** | 9 (53) | 32 (64) | 0.6 |
| **Age, in years, median (p25-75)** | 64 (59-72) | 59 (48-65) | 0.1 |
| **BMI, median (p25-75)** | 26 (23-28) | 25 (24-27) | 0.8 |
| **SOFA, median (p25-75)** | 5 (4-6) | 4 (3-5) | 0.03 |
| **APACHE II, median (p25-75)** | 21 (14-25) | 19 (14-25) | 0.8 |
| **Reason for admission, n (%)** | Medical 12 (71)  Surgical 5 (29) | Medical 26 (52)  Surgical 24 (48) | 0.3 |
| **Type of admission, n (%)** | Urgent 17 (100)  Scheduled 0 (0) | Urgent 46 (92)  Scheduled 4 (8) | 0.6 |
| **Comorbidities** | | | |
| **Hypertension, n (%)** | 5 (29) | 11 (22) | 0.5 |
| **Diabetes, n (%)** | 1 (6) | 6 (12) | 0.7 |
| **Chronic heart failure, n (%)** | 1 (6) | 1 (2) | 0.4 |
| **Chronic lung disease, n (%)** | 1 (6) | 1 (2) | 0.4 |
| **Asthma, n (%)** | 0 (0) | 1 (2) | 1 |
| **Chronic kidney disease, n (%)** | 1 (6) | 1 (2) | 0.4 |
| **Reintubation, n (%)** | 2 (12) | 4 (8) | 0.6 |
| **Ventilatory variables** | | | |
| **Hours with MP >18 J/min, median (p25-p75)** | 56 (6-222) | 7 (1-35) | 0.02 |
| **Hours with TV >8 ml/KgPBW, median median (p25-p75)** | 58 (39-218) | 25 (5-70) | 0.01 |
| **Hours with DP >15cmH2O, median median (p25-p75)** | 18 (6-242) | 5 (0.4-18) | 0.01 |

BMI = body mass index; SOFA = Sequential Organ Failure Assessment; APACHE = Acute Physiology and Chronic Health Evaluation; ICU= intensive care unit, MP = mechanical power; Vt = tidal volume; PBW = predicted body weight

e-Table S2. Univariate ICU mortality analysis. Mild hypoxemic patients

| **Variable** | **Died in the ICU**  (n=305) | **Survived in the ICU**  (n=954) | **P values** |
| --- | --- | --- | --- |
| **General characteristics and severity of the illness** | | | |
| **Male, n (%)** | 206 (67) | 630 (66) | 0.7 |
| **Age (years) median median (p25-p75)** | 68 (59-74) | 61 (48-71) | < 0.001 |
| **BMI, median median (p25-p75)** | 26 (24-29) | 26 (24-29) | 0.2 |
| **SOFA, median (p25-75)** | 6 (5-8) | 5 (3-6) | < 0.001 |
| **APACHE II, median (p25-75)** | 24 (19-29) | 19 (14-24) | < 0.001 |
| **Reason for admission, n (%)** | Medical 204 (67)  Surgical 101 (33) | Medical 525 (55)  Surgical 429 (45) | < 0.001 |
| **Type of admission, n (%)** | Urgent 296 (97)  Scheduled 9 (3) | Urgent 884 (93)  Scheduled 70 (7) | 0.01 |
| **Comorbidities** | | | |
| **Hypertension, n (%)** | 107 (35) | 241 (25) | 0.001 |
| **Diabetes, n (%)** | 44 (14) | 103 (11) | 0.1 |
| **Chronic heart failure, n (%)** | 14 (5) | 34 (4) | 0.5 |
| **Chronic lung disease, n (%)** | 15 (5) | 40 (4) | 0.7 |
| **Asthma, n (%)** | 3 (1) | 15 (2) | 0.6 |
| **Chronic kidney disease, n (%)** | 26 (8) | 28 (3) | < 0.001 |
| **Reintubation, n (%)** | 23 (8) | 87 (9) | 0.5 |
| **Ventilatory variables** | | | |
| **Hours with MP >18 J/min, median (p25-p75)** | 17 (4-61) | 22 (7-93) | 0.005 |
| **Hours with TV >8 ml/KgPBW, median (p25-p75)** | 62 (16-161) | 50 (18-135) | 0.4 |
| **Hours with DP >15cmH2O, median median (p25-p75)** | 26 (5-79) | 6 (1-41) | <0.001 |

BMI = body mass index; SOFA = Sequential Organ Failure Assessment; APACHE = Acute Physiology and Chronic Health Evaluation; ICU= intensive care unit, MP = mechanical power; Vt = tidal volume; PBW = predicted body weight

e-Table S3. Univariate ICU mortality analysis. Moderate hypoxemic patients

| **Variable** | **Died in the ICU**  **(n=408)** | **Survived in the ICU**  **(n=878)** | **P values** |
| --- | --- | --- | --- |
| **General characteristics and severity of illness** | | | |
| **Male, n (%)** | 296 (73) | 644 (73) | 0.8 |
| **Age (years), median (p25-75)** | 67 (58-74) | 63 (51-71) | < 0.001 |
| **BMI, median (p25-75)** | 28 (24-31) | 28 (25-31) | 0.1 |
| **SOFA, median (p25-75)** | 7 (5-8) | 5 (4-7) | < 0.001 |
| **APACHE II, median (p25-75)** | 23 (17-28) | 20 (14-25) | <0.001 |
| **Reason for admission, n (%)** | Medical 358 (88)  Surgical 50 (12) | Medical 732 (83)  Surgical 146 (17) | 0.05 |
| **Type of admission, n (%)** | Urgent 395 (97)  Scheduled 13 (3) | Urgent 857 (97)  Scheduled 24 (3) | 0.8 |
| **Comorbidities** | | | |
| **Hypertension, n (%)** | 162 (40) | 253 (29) | <0.001 |
| **Diabetes, n (%)** | 100 (24) | 130 (15) | <0.001 |
| **Chronic heart failure, n (%)** | 30 (7) | 38 (4) | 0.03 |
| **Chronic lung disease, n (%)** | 48 (12) | 43 (4) | <0.001 |
| **Asthma, n (%)** | 4 (1) | 14 (2) | 0.5 |
| **Chronic kidney disease, n (%)** | 44 (11) | 30 (3) | <0.001 |
| **Reintubation, n (%)** | 22 (5) | 73 (8) | 0.1 |
| **Ventilatory variables** | | | |
| **Hours with MP >18 J/min, median (p25-p75)** | 69 (24-223) | 61 (19-181) | 0.05 |
| **Hours with TV >8 ml/KgPBW, median (p25-p75)** | 60 (17-156) | 72 (23-188) | 0.01 |
| **Hours with DP >15cmH2O, median median (p25-p75)** | 46 (12-200) | 26 (5-118) | <0.001 |

BMI = body mass index; SOFA = Sequential Organ Failure Assessment; APACHE = Acute Physiology and Chronic Health Evaluation; ICU= intensive care unit, MP = mechanical power; Vt = tidal volume; PBW = predicted body weight

e-Table S4: Univariate ICU mortality analysis- Severe hypoxemic patients

| **Variable** | **Died in ICU**  (n=3) | **Survived in ICU**  (n=8) | **P Values** |
| --- | --- | --- | --- |
| **General characteristics and severity of illness** | | | |
| **Male, n (%)** | 3 (100) | 6 (75) | 1 |
| **Age (years), median (p25-75)** | 71 (69-76) | 56 (52-74) | 0.3 |
| **BMI, median (p25-75)** | 31 (28-35) | 31 (28-29) | 0.4 |
| **SOFA, median (p25-75)** | 5 (4-7) | 7 (5-10) | 0.5 |
| **APACHE II, median (p25-75)a** | 28 (23-33) | 19 (17-31) | 0.5 |
| **Reason for admission, n (%)** | Medical 3 (100)  Surgical 0 (0) | Medical 8 (100)  Surgical 0 (0) | - |
| **Type of admission, n (%)** | Urgent 3 (100)  Scheduled 0 (0) | Urgent 8 (100)  Scheduled 0 (0) | - |
| **Comorbidities** | | | |
| **Hypertension, n (%)** | 1 (33) | 2 (25) | 1 |
| **Diabetes, n (%)** | 1 (33) | 1 (12) | 0.4 |
| **Chronic heart failure, n (%)** | 0 (0) | 1 (12) | 1 |
| **Chronic lung disease, n (%)** | 1 (33) | 0 (0) | 0.3 |
| **Asthma, n (%)** | 0 (0) | 0 (0) | - |
| **Chronic kidney disease, n (%)** | 1 (33) | 0 (0) | 0.3 |
| **Reintubated, n (%)** | 1 (33) | 1 (12) | 0.5 |
| **Ventilatory variables** | | | |
| **Hours with MP>18 J/min, median (p25-p75)** | 183 (178-264) | 79 (65-147) | 0.1 |
| **Hours with TV >8ml/KgPBW, median (p25-p75)** | 106 (81-105) | 52 (11-105) | 0.3 |
| **Hours with DP >15cmH2O, median median (p25-p75)** | 41 (21-186) | 59 (26-112) | 0.84 |

BMI = body mass index; SOFA = Sequential Organ Failure Assessment; APACHE = Acute Physiology and Chronic Health Evaluation; ICU= intensive care unit, MP = mechanical power; Vt = tidal volume; PBW = predicted body weight

e-Figure S7. Pearson correlation hours with mechanical power >18 J/min and invasive mechanical ventilation days. Non-hypoxemic patients

*
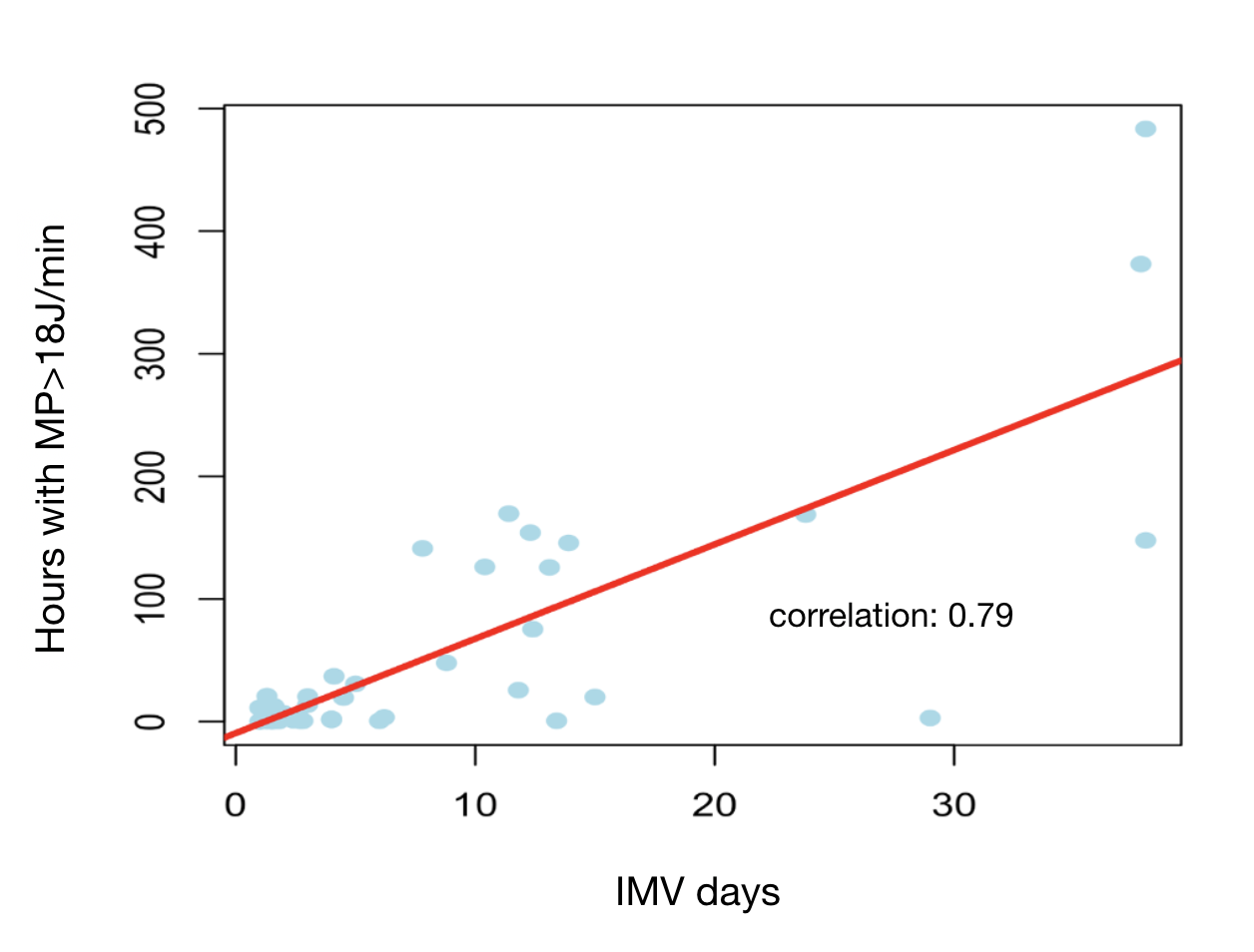
*

MP = mechanical power, IMV = invasive mechanical ventilation

e-Figure S8. Pearson correlation hours with mechanical power >18 J/min and invasive mechanical ventilation days. Mild hypoxemic patients

*
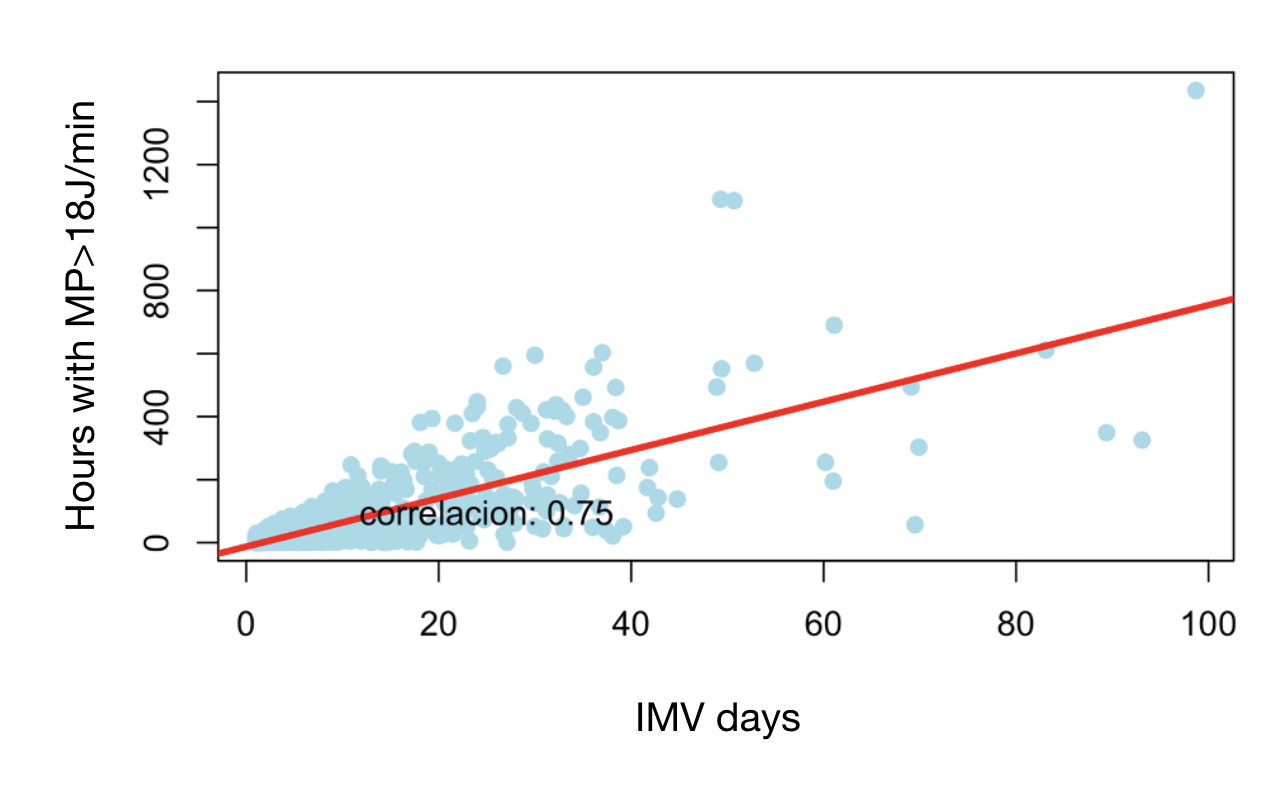
*

MP = mechanical power, IMV = invasive mechanical ventilation

e-Figure S9. Pearson correlation hours with mechanical power >18 J/min and invasive mechanical ventilation days. Moderate hypoxemic patients

*
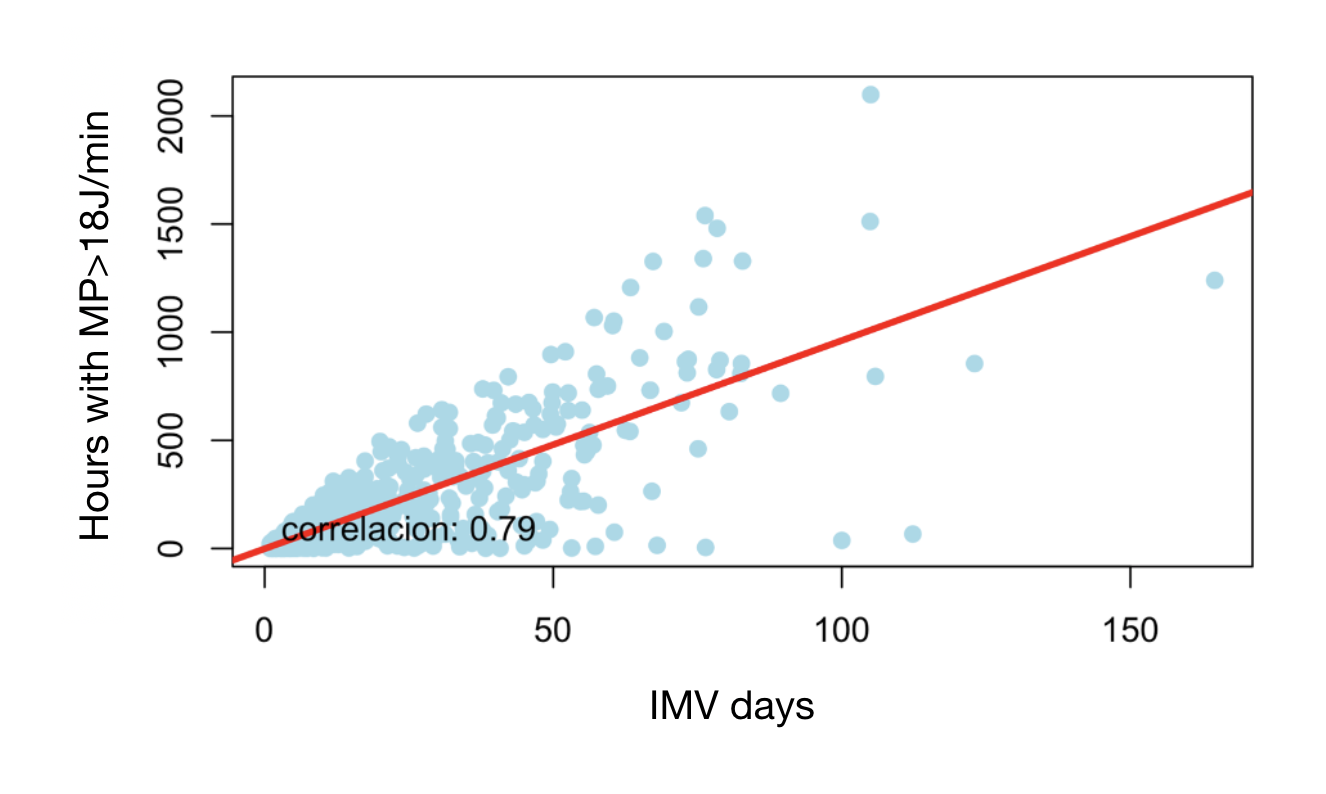
*

MP = mechanical power, IMV = invasive mechanical ventilation

e-Figure S10. Pearson correlation hours with mechanical power >18 J/min and invasive mechanical ventilation days. Severe hypoxemic patients.


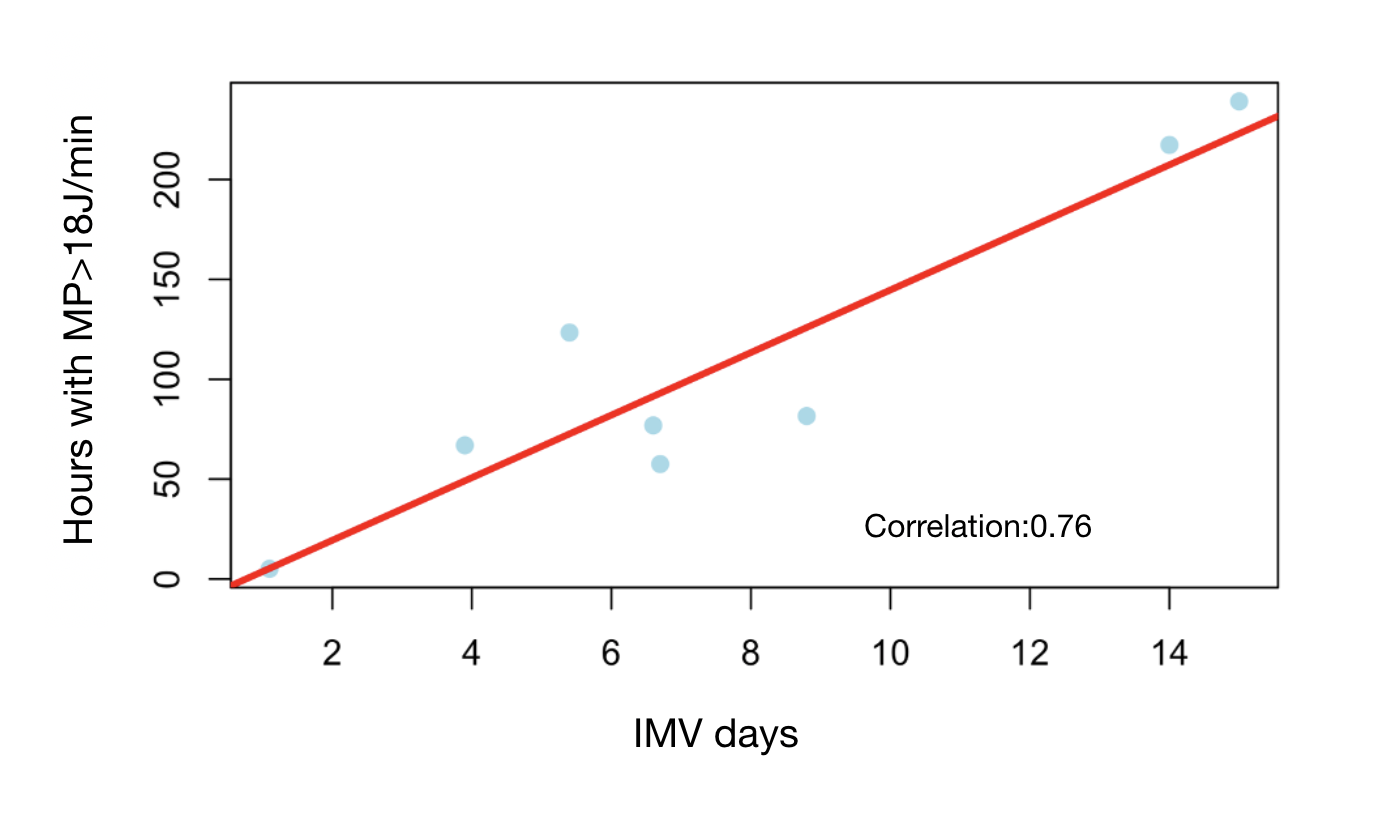


MP = mechanical power, ICU LOS = intensive care unit length of stay

e-Figure S11. Pearson correlation hours with mechanical power >18 J/min and intensive care unit length of stay. Non-hypoxic patients


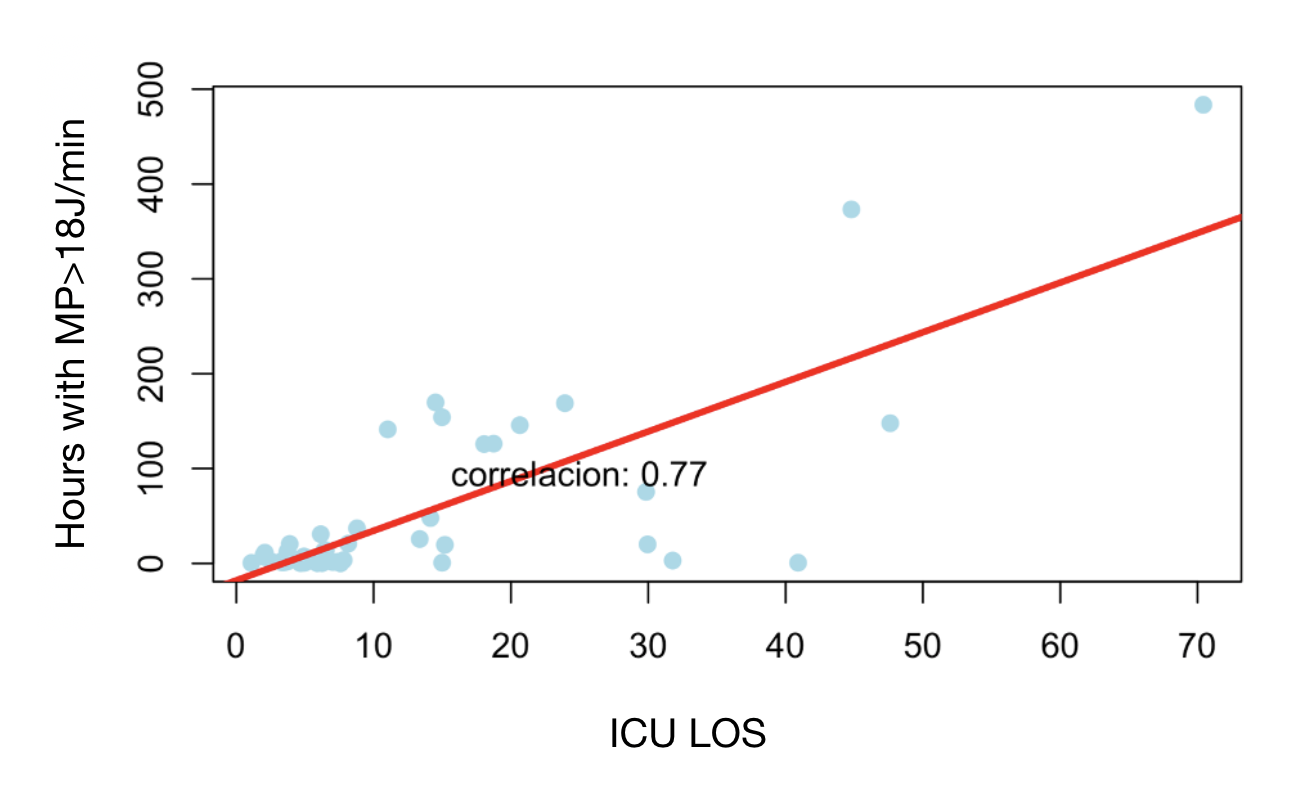


MP = mechanical power, ICU LOS = intensive care unit length of stay

e-Figure S12. Pearson correlation hours with mechanical power >18 J/min and intensive care unit length of stay. Mild hypoxic patients


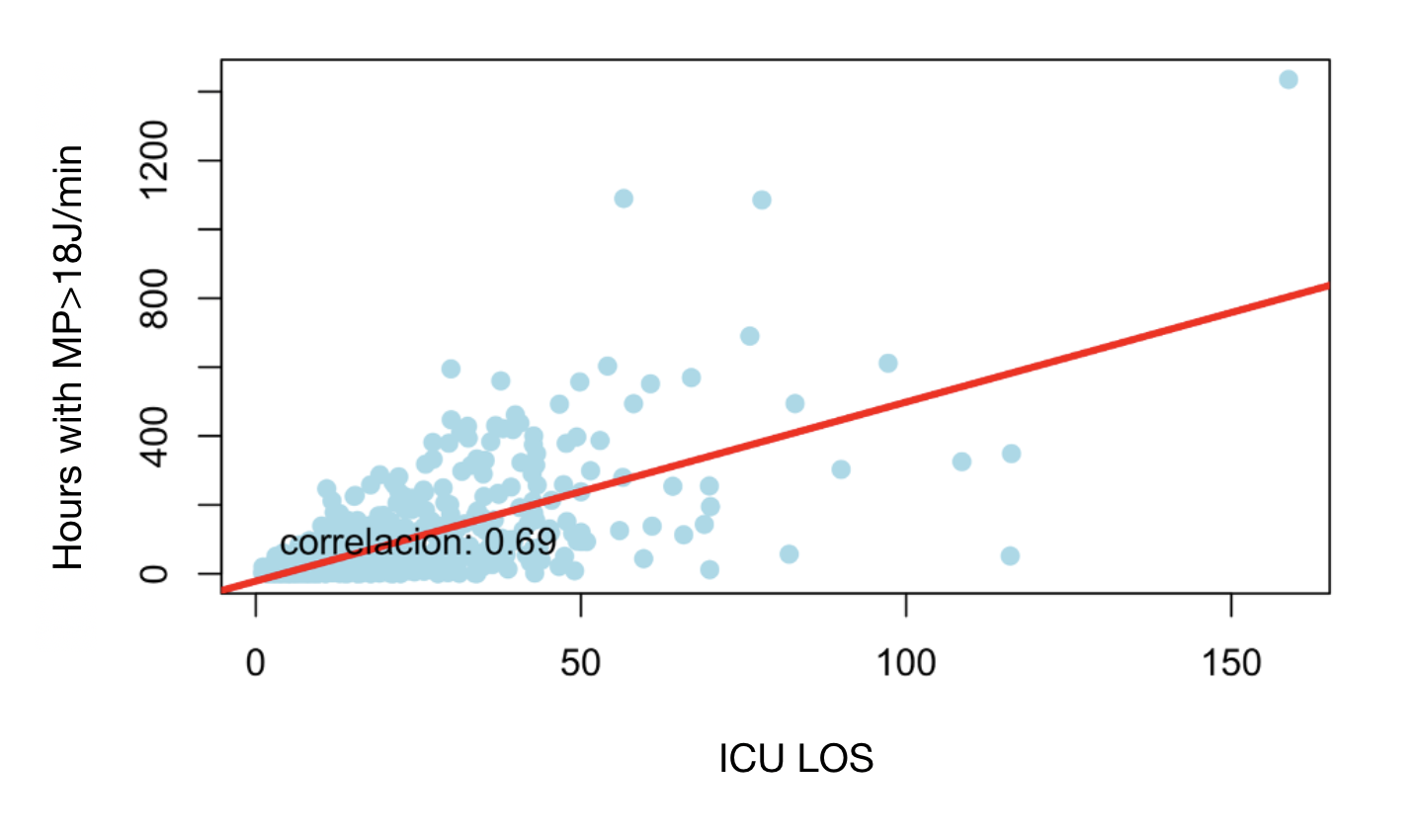


MP = mechanical power, ICU LOS = intensive care unit length of stay

e-Figure S13. Pearson correlation hours with mechanical power >18 J/min and intensive care unit length of stay. Moderate hypoxic patients


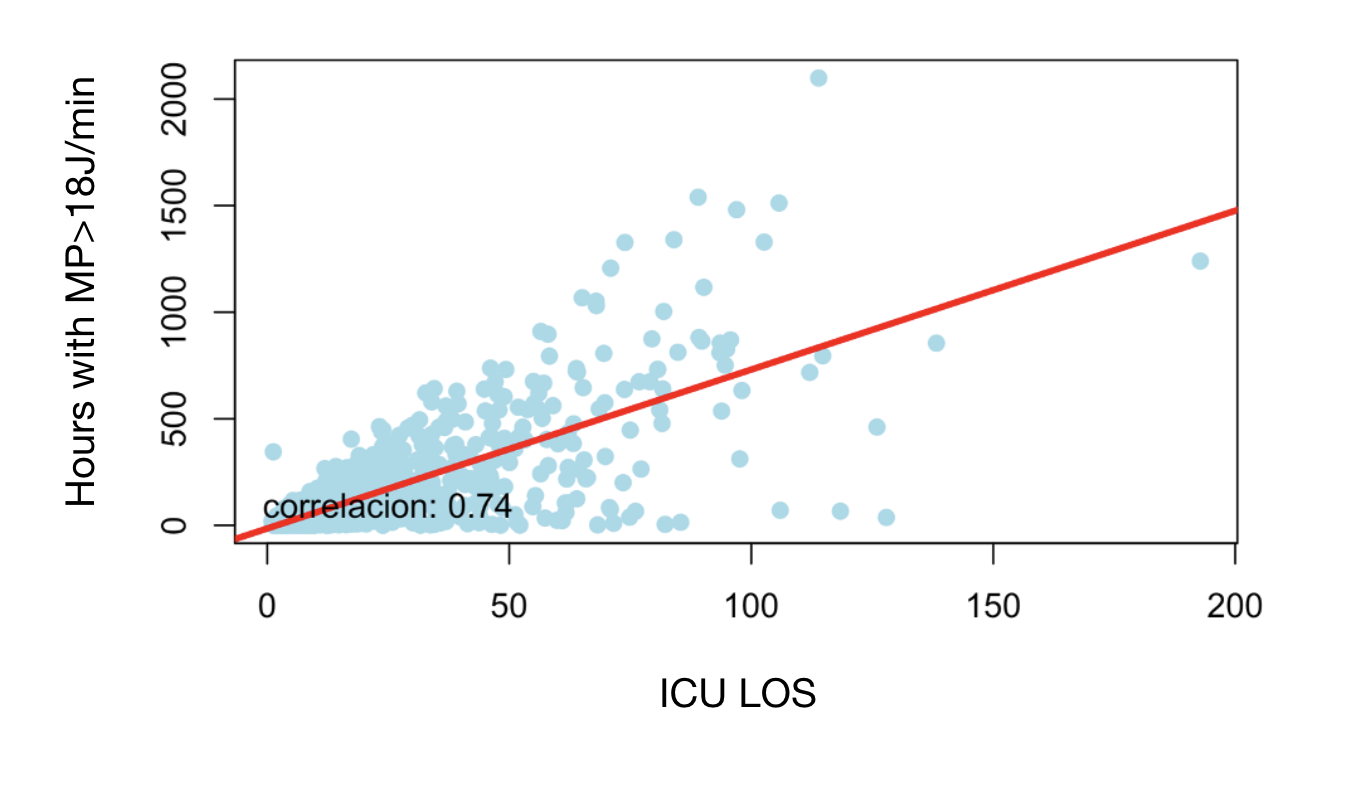


MP = mechanical power, ICU LOS = intensive care unit length of stay

e-Figure S14. Pearson correlation hours with mechanical power >18 J/min and intensive care unit length of stay. Severe hypoxic patients.


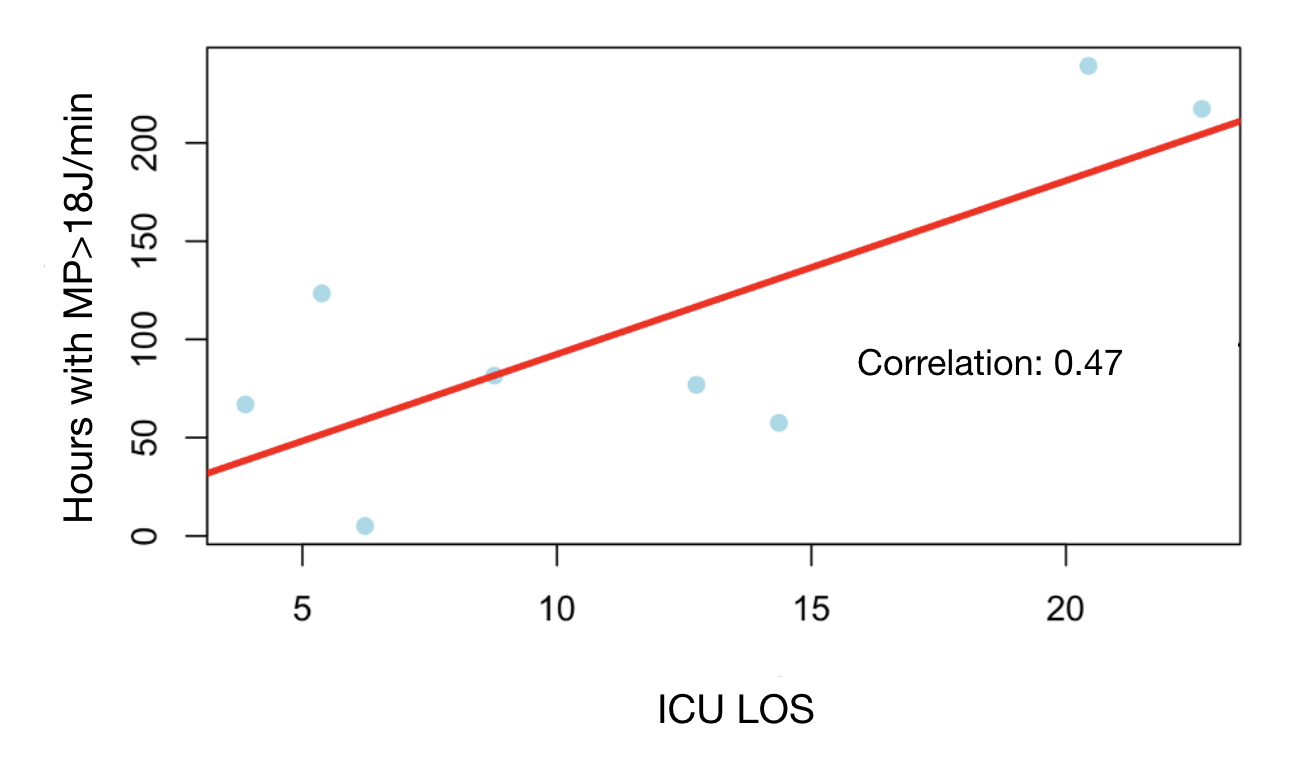


MP = mechanical power, ICU LOS = intensive care unit length of stay
